# SIRT1 promotes endothelial activity and osteogenesis *in vitro* and ameliorates age-related skeletal regeneration defects *in vivo*

**DOI:** 10.1016/j.stemcr.2026.103063

**Published:** 2026-09-03

**Authors:** Shibo Liu, Jingjing Shao, Qiheng Yang, Hanghang Liu, Yao Liu, Zizhuo Zheng, En Luo

**Affiliations:** 1 State Key Laboratory of Oral Diseases & National Center for Stomatology &National Clinical Research Center for Oral Diseases, West China Hospital of Stomatology, Sichuan University, Chengdu 610041, Sichuan, China; 2 MaineHealth Institute for Research, Maine Medical Center, Scarborough, ME 04074, USA; 3 Division of Oral Biology, Tufts University School of Dental Medicine, Boston, MA 02111, USA

**Keywords:** SIRT1, type H vessels, aging, osteoporosis, bone regeneration

## Abstract

Understanding age-driven bone weakening mechanisms is essential for developing precision therapeutic strategies. This study systematically investigated skeletal aging, focusing on the critical decline of SIRT1 and its impact on type H vessels and osteogenic cells. We found that SIRT1 markedly enhanced both endothelial tube formation and osteogenic differentiation in senescent bone marrow-derived mesenchymal stem cell (BMSC)/human umbilical vein endothelial cell (HUVEC) co-cultures. *In vivo* validation studies revealed that pharmacological activation of SIRT1 significantly improved bone regeneration in age-related osteoporotic defects in both femoral and mandibular sites. Mechanistically, β-catenin served as a downstream mediator of these effects in our experimental systems, with SIRT1 promoting β-catenin deacetylation and nuclear translocation to activate Wnt signaling. By demonstrating that SIRT1 enhances both endothelial and osteogenic functions in the aged bone microenvironment, this work provides a rationale for targeted bone rejuvenation strategies and mechanistically informed therapeutic innovation against geriatric osteoporosis.

## Highlights

• SIRT1 activation enhances endothelial and osteogenic functions in aged bone
• Wnt/β-catenin signaling mediates SIRT1’s effects on both lineages
• Pharmacological SIRT1 targeting improves age-related bone regeneration

## Graphical abstract

**Figure:**
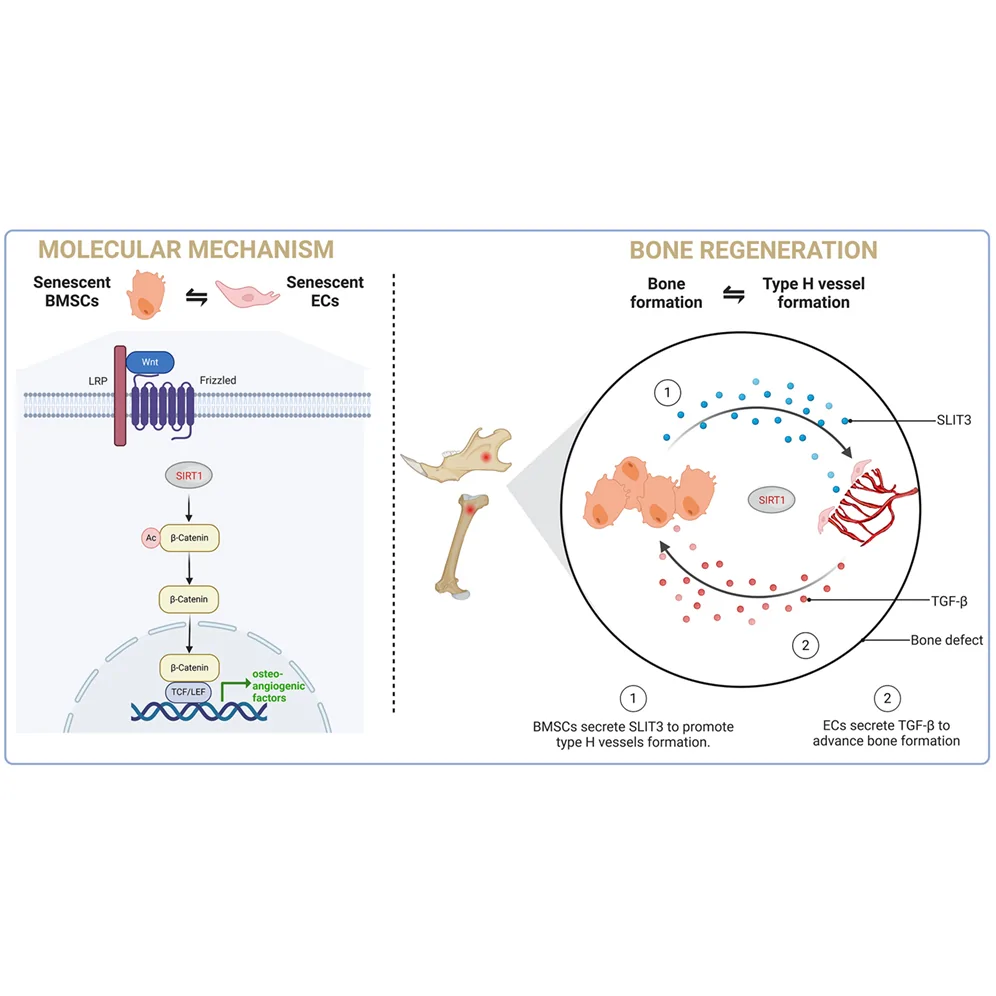

## Teaser

In this article, Luo and colleagues show that SIRT1 activation counteracts bone aging by enhancing endothelial and osteogenic functions in senescent BMSCs via β-catenin deacetylation. SIRT1 rejuvenates type H vessels and improves osteoporotic bone regeneration, establishing it as a therapeutic target for geriatric osteoporosis.

## Introduction

Aging constitutes a multisystem degenerative process marked by progressive musculoskeletal deterioration, with osteoporosis emerging as a pathological hallmark characterized by systemic bone mass reduction affecting both cortical and trabecular compartments (Sözen et al., 2017). This structural decay heightens fracture risk while delaying bone repair. Clinically, these alterations manifest as heightened morbidity-mortality rates and profound quality-of-life deterioration in geriatric populations, while simultaneously imposing staggering socioeconomic burdens on healthcare systems grappling with global demographic aging (Boz and Ozsarı, 2020; Guo et al., 2021). Consequently, understanding age-driven bone weakening mechanisms is key to governing bone homeostasis to develop precision therapeutic strategies for skeletal regeneration.

The skeletal system maintains intricate vascular networks that dynamically regulate bone formation and metabolic homeostasis. Central to this regulation is the spatiotemporal coordination of angiogenesis and osteogenesis, a process whose dysregulation underlies bone pathologies (Lafage-Proust et al., 2010; Saran et al., 2014). Recent advances have identified type H vessels—CD31^hi^/EMCN^hi^ endothelial structures concentrated at metaphyseal and periosteal regions—as metabolic hubs critical for maintaining local oxygen tension, hemodynamics, and nutrient delivery to support osteogenesis (Kusumbe et al., 2014, 2016). These vessels form functional niches with OSTERIX+/RUNX2+/COL1+ osteoprogenitors, serving dual roles as osteoblast reservoirs and secretors of pro-osteogenic factors (PDGFs, FGF1, and TGFs) that drive osteoprogenitor expansion and differentiation (Peng et al., 2020). Mechanistically, bidirectional signaling between bone lining cells and endothelia sustains this vascular-osteogenic circuitry, whose breakdown correlates with skeletal degeneration (Chen et al., 2020a; Wu et al., 2020; Xu et al., 2018). Crucially, aging markedly reduces type H vessel density and associated osteoprogenitors in long bones (Kusumbe et al., 2014; Ramasamy et al., 2016), though pan-skeletal mapping of these age-related vascular changes remains incomplete. Furthermore, the pathophysiological interplay between senescent bone marrow-derived mesenchymal stem cells (BMSCs) and endothelia—a hallmark of skeletal aging—and its metabolic consequences demand systematic investigation to unravel therapeutic targets for age-related bone loss.

As the most evolutionarily conserved and functionally versatile member of the sirtuin family (SIRT1-7), SIRT1 distinguishes itself through unique structural attributes and NAD+-dependent deacetylase activity (Chang and Guarente, 2014). This epigenetic regulator orchestrates cellular adaptation to stressors by modulating transcriptional landscapes and post-translational modifications, thereby governing critical processes spanning senescence attenuation, redox homeostasis, and inflammatory resolution (Luo et al., 2022; Singh and Ubaid, 2020). In skeletal contexts, SIRT1 exerts multifaceted governance over bone-vascular cross-regulation—enhancing osteogenesis through reprogramming BMSCs’ functions while preserving vascular integrity via endothelial migration potentiation (Chen et al., 2020b; Wang et al., 2014). Notably, its reactive oxygen species (ROS)-quenching capacity reverses oxidative damage-induced osteogenic impairment in osteoporotic BMSCs, suggesting therapeutic potential. However, whether SIRT1 regulates type H vessel density or mediates the functional relationship between aged BMSCs and endothelial cells remains largely unknown. Moreover, the molecular pathways through which SIRT1 exerts these potential effects in the aged bone microenvironment require further elucidation.

This investigation undertook systematic mapping of skeletal aging trajectories spanning long bones and jaw structures, mechanistically implicating SIRT1 as a key regulator of senescent type H vessel and BMSC function. By elucidating its role in orchestrating the endothelial and osteogenic functions, this study aims to provide a rationale for targeted bone rejuvenation strategies and mechanistically informed therapeutic innovation against geriatric osteoporosis.

## Results

### SIRT1 alleviated age-related osteoporosis in association with type H vessels and osteogenic function

Micro computed tomography (micro-CT) volumetric analysis demonstrated significant bone loss in aged mice, with structural degradation evident in femur (Figure 1A). Quantifiable trabecular architectural impairments included decreased bone volume/total volume (BV/TV), diminished trabecular number (Tb.N) and trabecular thickness (Tb.Th), and increased trabecular separation (Tb.Sp) compared with young controls (Figure 1B). Senescence-associated β-galactosidase (SA-β-gal) activity staining showed an increase in senescent cell burden within aged skeletal tissues (Figure 1A). The density of type H vasculature demonstrates gradual attenuation across skeletal aging trajectories (Figure 1A). The tibia and mandible showed similar intergroup differences. (Figure S1).

**Figure 1:**
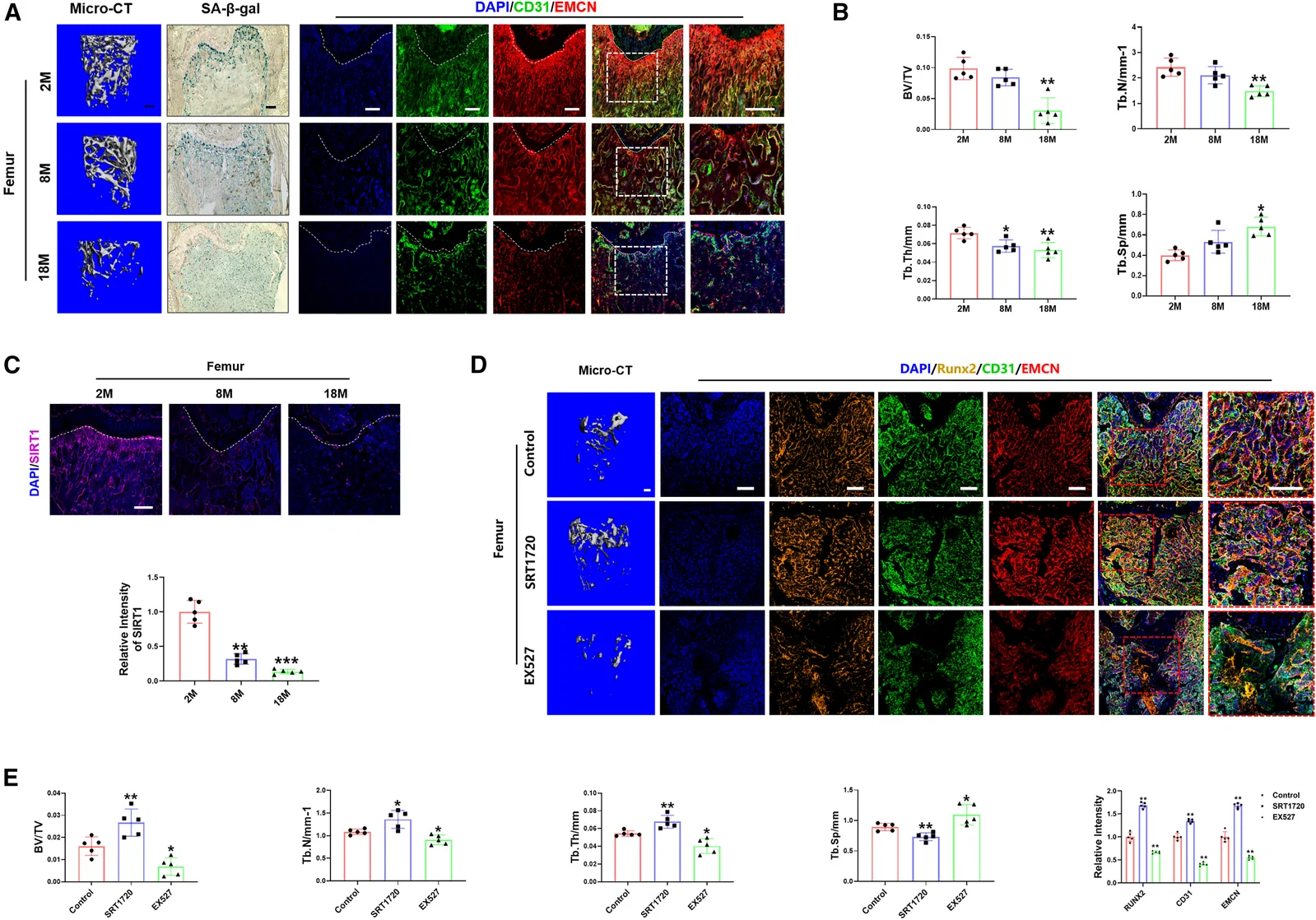
SIRT1 alleviated age-related osteoporosis in association with type H vessels and osteogenic function (A) Micro-CT volumetric reconstructions, SA-β-gal staining, and immunofluorescence co-staining of CD31^hi^EMCN^hi^-type H vessels across aging cohorts femoral bones. (B) Correlative morphometric analyses of micro-CT reconstructed images. (C) Age-stratified immunofluorescence staining of SIRT1 expression gradients in femoral osseous compartments. (D and E) Type H vessel, osteogenic activity, and micro-CT reconstructions with correlative morphometric quantification in the femoral bone of aged mice (18 months old) following 8-week intraperitoneal administration of SRT1720 and EX527. Scale bars, 200 μm. Data presented as mean ± SD (*n* = 5 mice per group). For each biological replicate, technical triplicates were measured and averaged; ^∗^*p* < 0.05, ^∗∗^*p* < 0.01, and ^∗∗∗^*p* < 0.001 by one-way ANOVA with Tukey’s post hoc test.

Immunofluorescence analysis revealed a progressive age-dependent reduction in SIRT1 expression of femur (Figure 1C). To investigate the therapeutic potential of SIRT1 modulation, osteoporotic male mice (18 months old) received either SRT1720 or EX527 administration. Pharmacological activation of SIRT1 with SRT1720 significantly enhanced the abundance of type H vessels and their associated RUNX2+ osteoprogenitor cells after 8-week treatment (Figures 1D and 1E). Micro-CT analysis demonstrated substantial improvement in bone microstructure following SRT1720 administration, characterized by increased trabecular number, enhanced trabecular density, and thickened trabecular morphology compared with age-matched controls (Figure 1D). Quantitative morphometric parameters confirmed these observations, showing significant increases in BV/TV, Tb.N, and Tb.Th, accompanied by reduced Tb.Sp (Figure 1E). Conversely, SIRT1 inhibition with EX527 exacerbated age-related bone loss and reduced type H vessel density and osteoprogenitor association (Figures 1D and 1E). Regulation of SIRT1 induced similar bone-vascular phenotypic changes in the tibia (Figure S2).

### SIRT1 enhances osteogenic and angiogenic capacities in senescent cells *in vitro*

Oxidative stress-induced senescence modeling in BMSCs and human umbilical vein endothelial cells (HUVECs) was validated through H_2_O_2_ exposure, showing concomitant upregulation of senescence-associated markers (p53, p16, and p21) and elevated SA-β-gal+ cell prevalence (Figures 2A–2C and 2I–2K). Functional characterization revealed concurrent impairment of osteogenic differentiation capacity, evidenced by reduction in alkaline phosphatase (ALP) activity, alizarin red S (ARS)-stained mineralization (Figure 2A) and decrease in tube formation (Figures 2I and S3).

**Figure 2:**
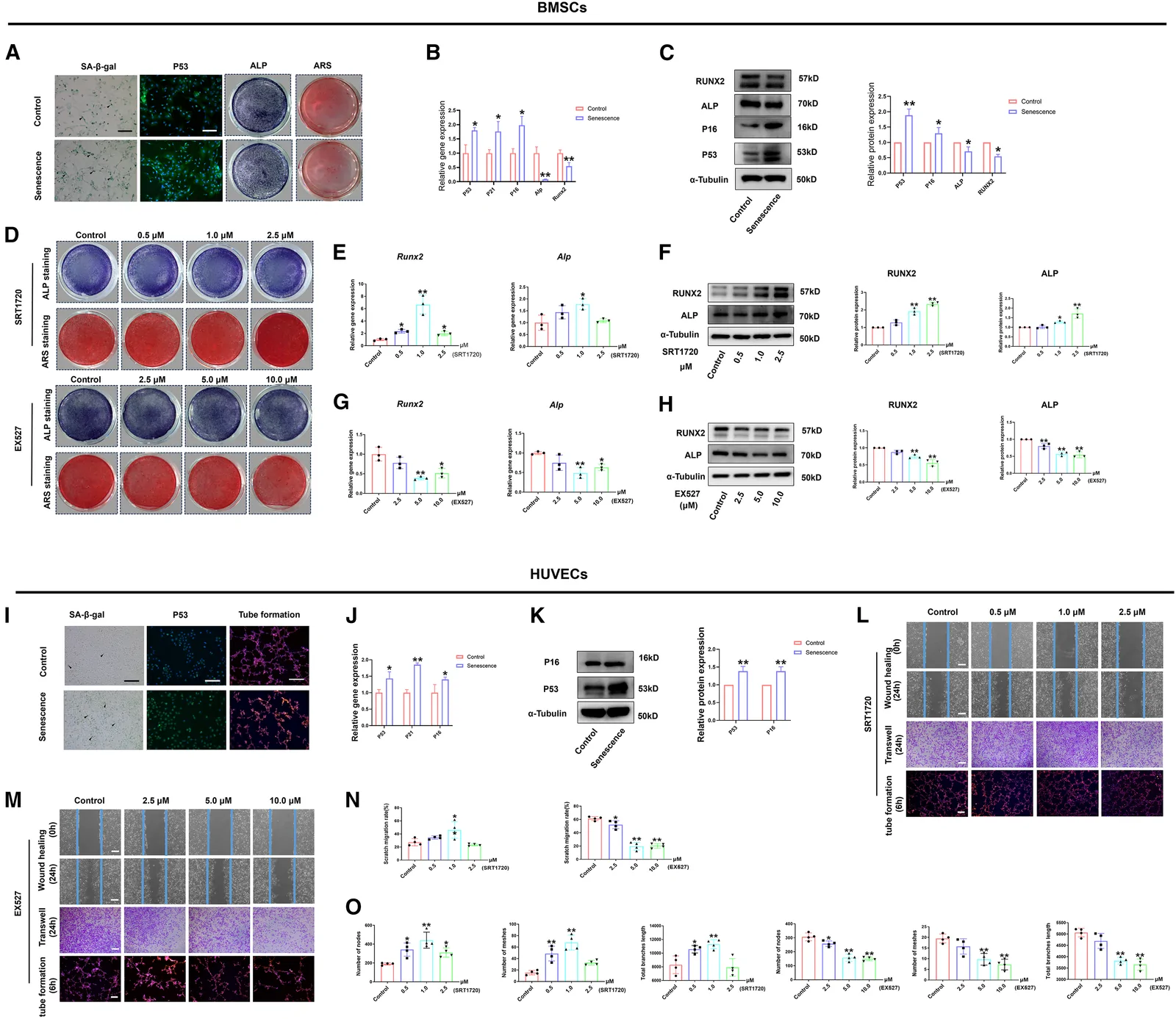
Pharmacological SIRT1 activation improves age-compromised osteogenic and angiogenic efficiency *in vitro* (A) SA-β-gal staining, p53 protein staining, and ALP and ARS staining in young versus senescent BMSCs. Scale bars, 200 μm. (B and C) Senescence marker expression and osteogenic marker expression in BMSCs assessed by qRT-PCR (B) and western blot (C). (D) SIRT1 activation (SRT1720) and inhibition (EX527) regulated the osteogenic potential of senescent BMSCs as demonstrated by ALP and ARS staining. (E–H) qRT-PCR and western blot analyses of osteo-lineage markers in senescent BMSCs following 7-day osteogenic induction. *n* = 3 independent experiments. (I) SA-β-gal staining, p53 protein staining, tube formation assay, and quantitative analysis in HUVECs. Scale bars, 200 μm. (J and K) Senescence marker expression in HUVECs assessed by qRT-PCR (J) and western blot (K). (L and M) SIRT1 activation (SRT1720) and inhibition (EX527) regulated angiogenic competence in senescent HUVECs as evidenced by wound-healing, transwell migration, and tube formation assays. Scale bars, 100 μm. (N) Quantified scratch closure results. *n* = 4 independent HUVEC cultures. (O) Quantitative analysis results of branching nodes, meshes and length in tube formation assay. *n* = 4 independent HUVEC cultures. Data presented as mean ± SD from the indicated number (*n*) of biologically independent experiments or cell culture preparations; ^∗^*p* < 0.05, and ^∗∗^*p* < 0.01, by one-way ANOVA with Tukey’s post hoc test.

In senescence-induced BMSCs, functional assessments confirmed these molecular changes, with SRT1720-treated cells showing enhanced ALP activity and increased calcium nodule deposition compared with untreated senescent controls (Figures 2D and S3). Both quantitative real-time PCR and western blot analyses demonstrated that SRT1720-mediated SIRT1 activation markedly upregulated early osteogenic markers (ALP and RUNX2) following 7 days of osteogenic induction (Figures 2E and 2F). Conversely, pharmacological inhibition of SIRT1 with EX527 caused marked suppression of these critical osteogenic parameters (Figures 2D, 2G, 2H, and S3). Similar results were observed in replicatively senescent BMSCs (passage 8; Figure S4).

Parallel experiments in senescent HUVECs revealed that SIRT1 activation significantly improved cellular migration capacity, as evidenced by scratch wound closure assays and transwell migration analyses (Figures 2L and 2N). Tube formation assays further demonstrated SIRT1’s pro-angiogenic role, with SRT1720-treated HUVECs exhibiting enhanced capillary-like network formation (Figures 2L and 2O). In contrast, EX527 administration substantially impaired both migratory and tube-forming capacities of senescent HUVECs (Figures 2M–2O).

### SIRT1 enhances bidirectional paracrine effects between senescent BMSCs and senescent HUVECs via the Wnt/β-catenin signaling axis

To delineate the mechanism underlying SIRT1-mediated paracrine effects between BMSCs and HUVECs, a bidirectional co-culture system was established between BMSCs and HUVECs (Figure 3A). It should be noted at the outset that this conditional medium (CM) system specifically reflects soluble factor-mediated effects and does not assess direct, contact-dependent cellular interactions. CM from SRT1720-activated senescent HUVECs exhibited elevated secretion of transforming growth factor-β (TGF-β), a potent osteogenic inducer (Figure 3B), correlating with enhanced osteogenic differentiation capacity (Figures 3C and 3D). Reciprocally, SRT1720-treated senescent BMSCs secreted increased levels of SLIT homolog 3 (SLIT3), a critical mediator of type H vessel formation, as quantified by ELISA (Figure 3E). Functional validation revealed that CM from SIRT1-activated BMSCs significantly potentiated senescent HUVEC migration and tube formation compared with control CM (Figures 3F–3I). EX527 exerted an opposite effect on these paracrine effects (Figure 3).

**Figure 3:**
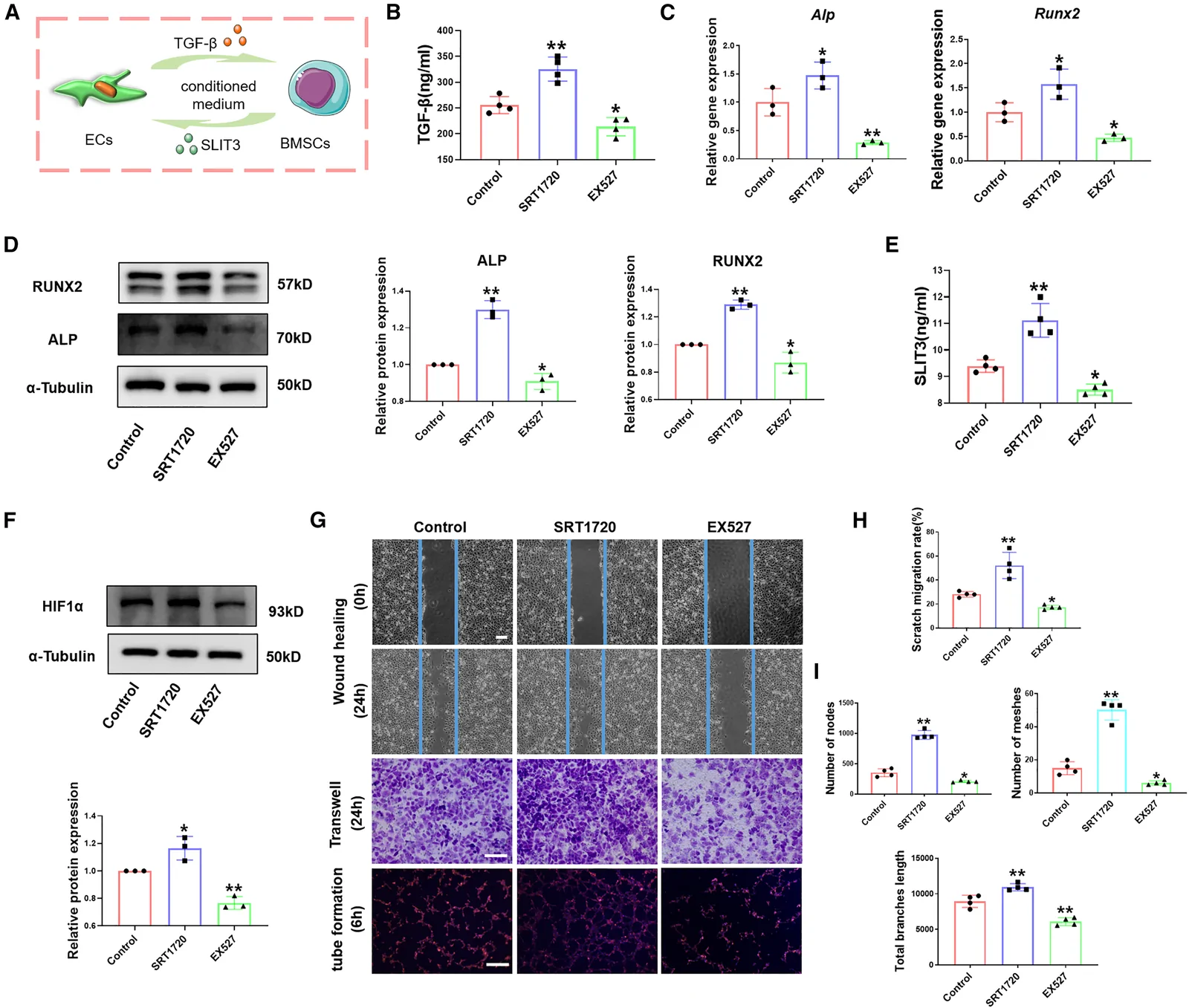
SIRT1 enhances bidirectional paracrine effects between senescent BMSCs and HUVECs (A) Schematic diagram illustrating the bidirectional paracrine effects established by senescent BMSCs and HUVECs. (B) ELISA quantification of TGF-β secretion levels from senescent HUVECs. *n* = 4 independent senescent HUVEC cultures. (C and D) qRT-PCR and western blot analyses of senescent BMSCs treated with conditional media from senescent HUVECs. *n* = 3 independent experiments. (E) ELISA quantification of SLIT3 secretion levels from senescent BMSCs. *n* = 4 independent senescent BMSC cultures. (F) Western blot analysis of HUVECs treated with conditional media from senescent BMSCs. *n* = 3 independent experiments. (G–I) Representative images and quantitative analysis of HUVEC functional assessments (wound healing, transwell migration, and tube formation assays) following treatment with BMSC-conditional media. *n* = 4 independent experiments. Scale bars, 100 μm. Data presented as mean ± SD from the indicated number (*n*) of biologically independent experiments or cell culture preparations; ^∗^*p* < 0.05, ^∗∗^*p* < 0.01 by one-way ANOVA with Tukey’s post hoc test.

RNA sequencing (RNA-seq) results identified age-associated dysregulation of the Wnt signaling pathway, between young and aged cohorts (Figure 4A). Immunofluorescence confirmed progressive age-related depletion of β-catenin in skeletal tissues (Figure 4B). Mechanistically, SRT1720 promoted nuclear accumulation of β-catenin in both senescent BMSCs and HUVECs, concomitant with upregulation of downstream Wnt targets (Figures 4C–4F). These effects were reversed by EX527, confirming SIRT1-dependent regulation of β-catenin signaling.

**Figure 4:**
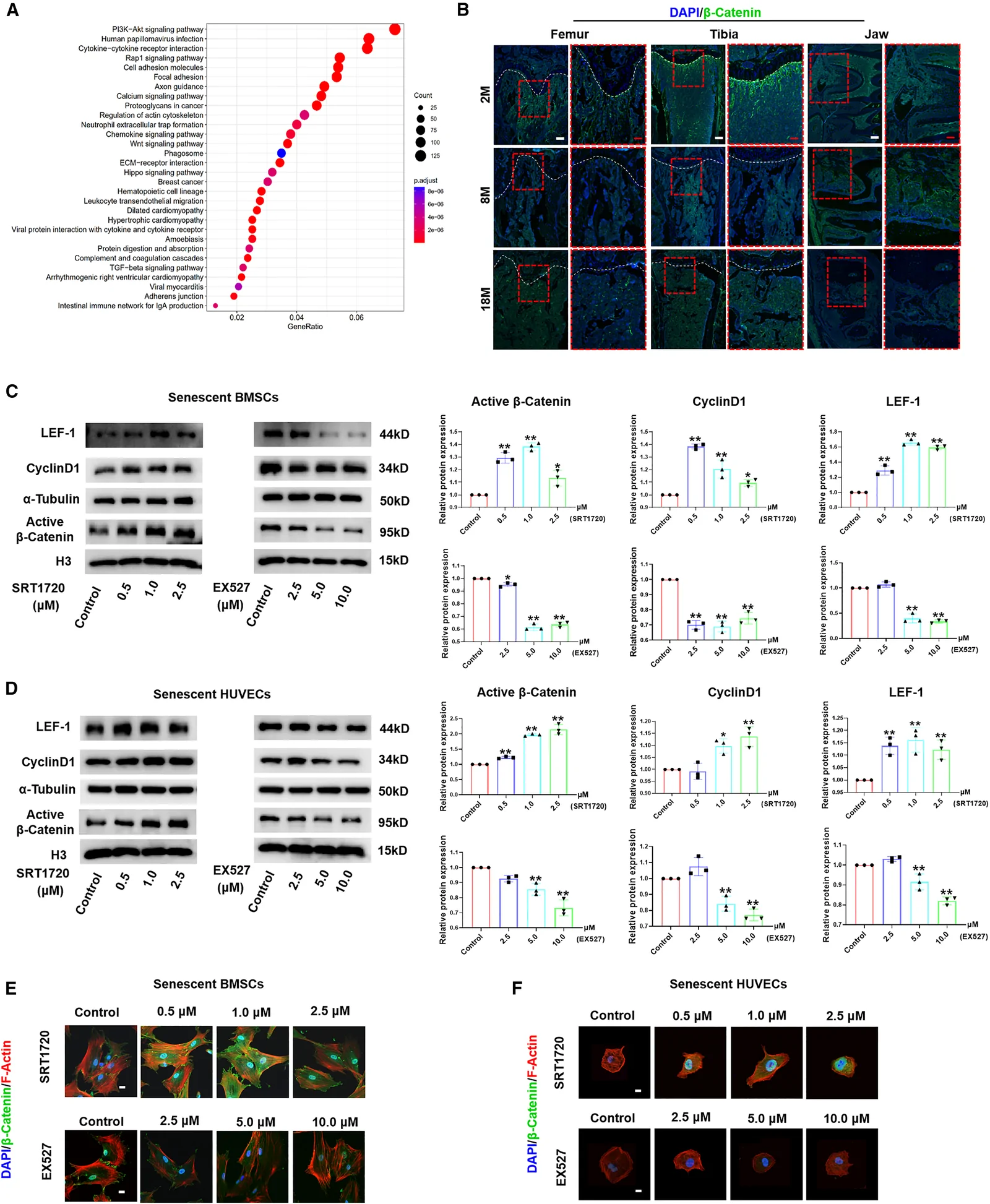
SIRT1 regulates senescent BMSCs and HUVECs through the Wnt/β-catenin signaling pathway (A) Gene Ontology (GO) enrichment analyses of endothelial cells isolated from young versus aged mice. (B) IF staining of the Wnt pathway key effector β-catenin in murine jaw, femur, and tibia across different age groups. Scale bars: 100 μm (white), 50 μm (red). *n* = 5 mice per group. (C and D) Western blot and quantitative analysis of Wnt signaling components in senescent BMSCs (C) and senescent HUVECs (D) after SRT1720 and EX527 treatment. *n* = 3 independent experiments. (E and F) IF staining of β-catenin in senescent BMSCs (E) and HUVECs (F) demonstrating nuclear translocation. *n* = 3 independent experiments. Scale bars, 20 μm. Data presented as mean ± SD from the indicated number (n) of biologically independent experiments; ^∗^*p* < 0.05, ^∗∗^*p* < 0.01 by one-way ANOVA with Tukey’s post hoc test.

The validation of β-catenin knockdown efficiency in senescent BMSCs and HUVECs is presented in Figure S5. Gene knockdown of *Ctnnb1* (encoding β-catenin) weakened SRT1720-induced enhancements in osteogenic and angiogenic functions (Figures 5A and 5C). Crucially, *Ctnnb1* knockdown attenuated the paracrine crosstalk between senescent cells, reducing both TGF-β secretion from HUVECs (Figure 5B) and SLIT3 production from BMSCs (Figure 5D). This bidirectional disruption of paracrine signaling (Figures 5E and 5F) establishes β-catenin as the essential mediator of SIRT1’s pro-regenerative effects in aged skeletal niches.

**Figure 5:**
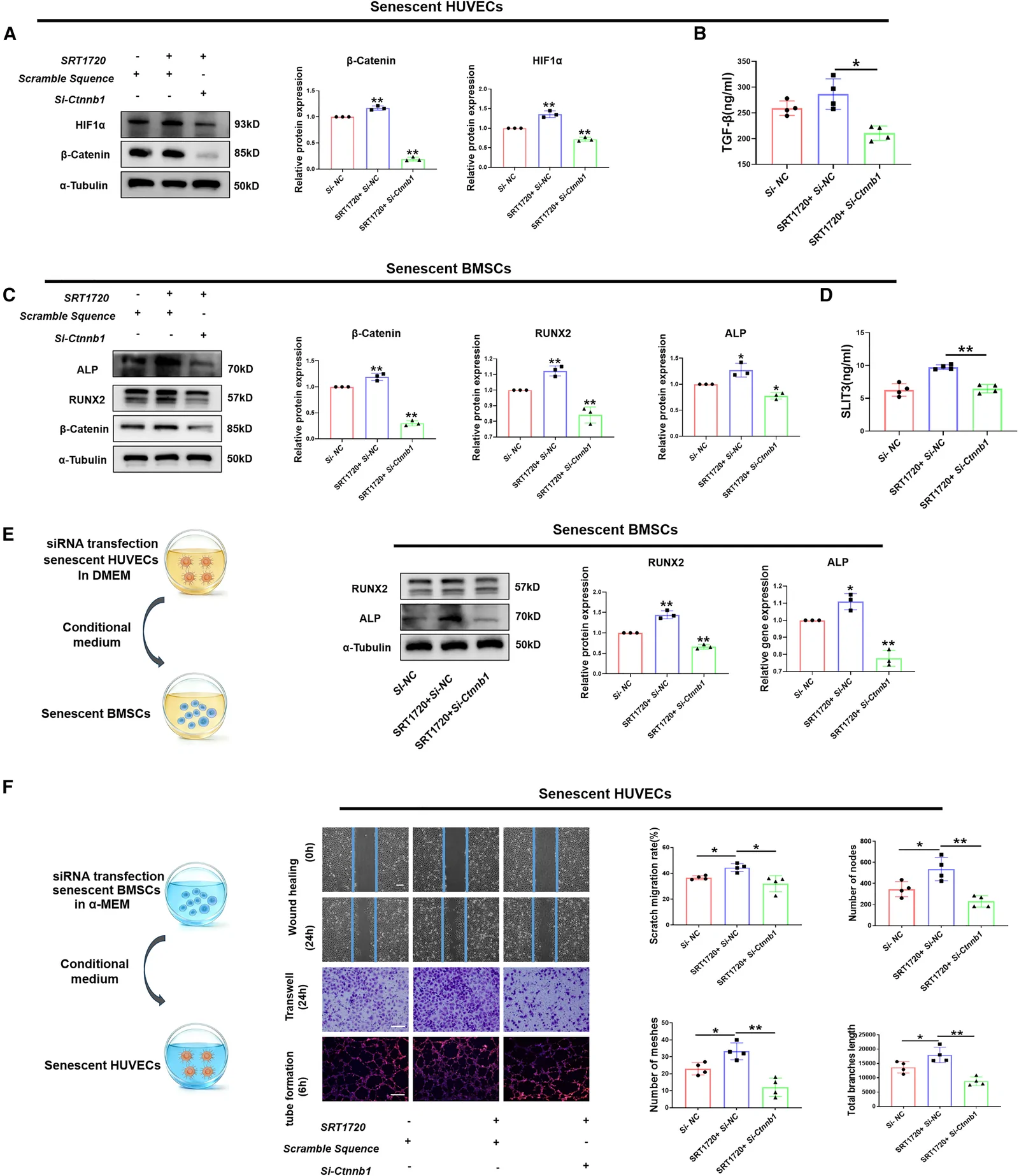
SIRT1 promotes bidirectional paracrine effects between senescent BMSCs and HUVECs through a β-catenin-mediated pathway (A) Western blot and quantitative analysis of angiogenic regulators in senescent HUVECs following *Ctnnb1* knockdown. *n* = 3 independent experiments. (B) ELISA quantification of TGF-β secretion from senescent HUVECs post-*Ctnnb1* knockdown. *n* = 4 independent senescent HUVEC cultures. (C) Osteogenic differentiation capacity of *Ctnnb1*-knockdown senescent BMSCs assessed by western blot. *n* = 3 independent experiments. (D) ELISA quantification of SLIT3 secretion from *Ctnnb1*-knockdown senescent BMSCs. *n* = 4 independent senescent BMSC cultures. (E) Western blot and quantitative analysis of osteogenic markers in senescent BMSCs treated with conditional media from *Ctnnb1*-knockdown senescent HUVECs. *n* = 3 independent experiments. (F) Functional validation of senescent HUVECs (wound healing, transwell migration, and tube formation) treated with senescent BMSC-conditional media after *Ctnnb1* knockdown. *n* = 4 independent experiments. Scale bars, 100 μm. Data presented as mean ± SD from the indicated number (*n*) of biologically independent experiments or cell culture preparations; ^∗^*p* < 0.05, ^∗∗^*p* < 0.01 by one-way ANOVA with Tukey’s post hoc test.

### SIRT1 coordinates β-catenin regulation through transcriptional activation and post-translational deacetylation

To elucidate SIRT1’s molecular regulation of β-catenin, we systematically interrogated its effects across gene expression, protein synthesis, and post-translational modification axes. Transcriptional profiling via qRT-PCR and translational assessment by western blot revealed that SIRT1 activation with SRT1720 significantly upregulated *Ctnnb1* mRNA and β-catenin protein levels compared with untreated controls, while EX527-mediated SIRT1 inhibition suppressed both expression tiers (Figures 6A–6C). Co-immunoprecipitation (coIP) assays confirmed direct physical interaction between SIRT1 and β-catenin, suggesting functional cooperativity (Figure 6D). Acetylation of β-catenin was assessed globally using a pan-acetyl-lysine antibody following immunoprecipitation. SRT1720 treatment reduced total β-catenin acetylation levels compared to controls, whereas EX527 increased them (Figures 6E and 6F), confirming that SIRT1 functions as a deacetylase for β-catenin. This inverse relationship between SIRT1 activity and β-catenin acetylation positions SIRT1 as a critical deacetylase governing β-catenin’s functional state.

**Figure 6:**
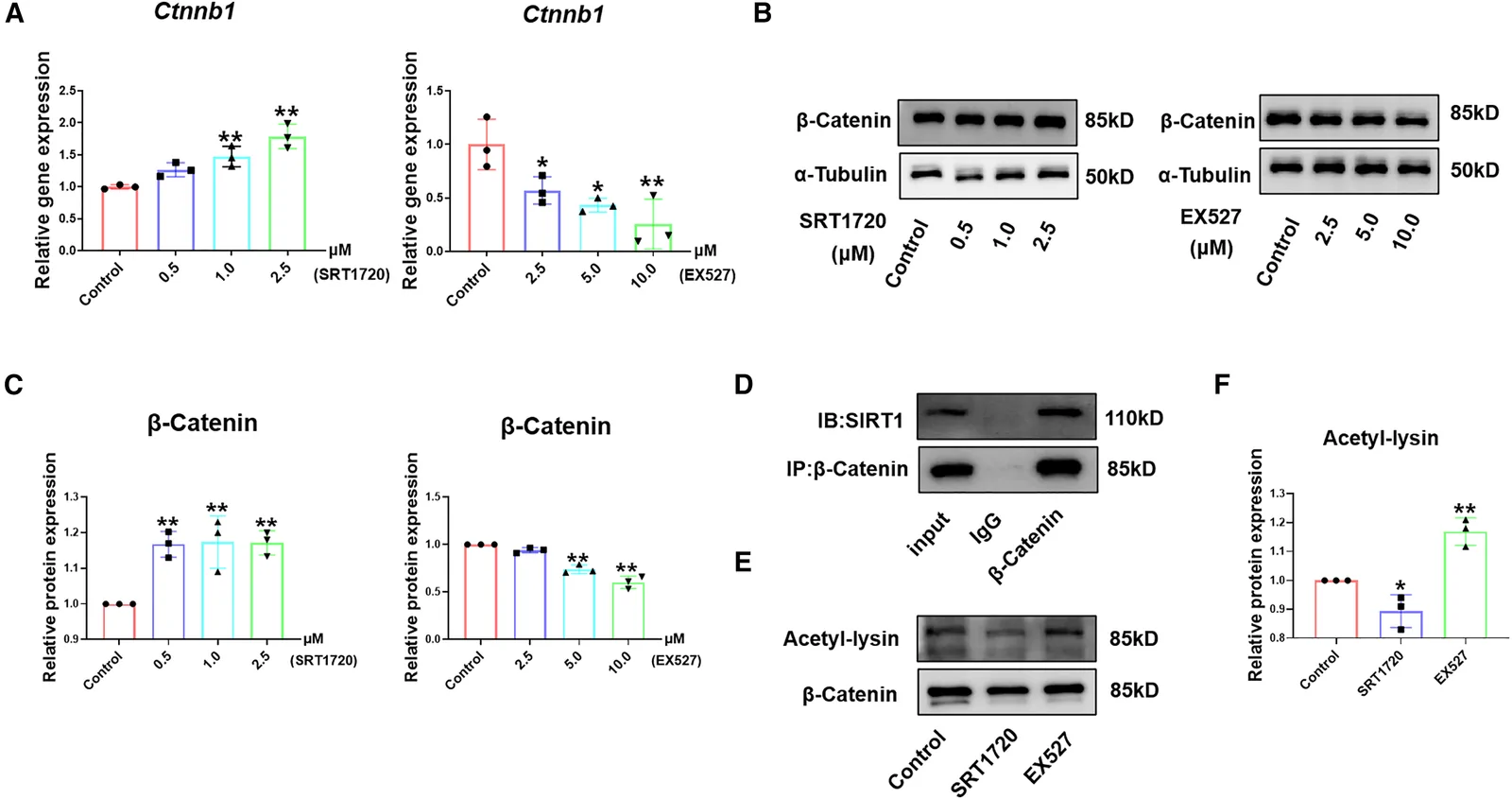
Mechanistic exploration of SIRT1-mediated β-catenin regulation (A) qRT-PCR analysis of β-catenin mRNA levels under SIRT1 functional modulation. *n* = 3 independent experiments. (B and C) Western blot and quantitative analysis of β-catenin protein expression regulated by SIRT1. *n* = 3 independent experiments. (D) Co-immunoprecipitation (coIP) assay confirming direct SIRT1-β-catenin binding. *n* = 3 independent experiments. (E and F) Deacetylation assay after immunoprecipitation of β-catenin and quantitative analysis demonstrating SIRT1-dependent β-catenin deacetylation. *n* = 3 independent experiments. Data presented as mean ± SD from the indicated number (*n*) of biologically independent experiments; ^∗^*p* < 0.05, ^∗∗^*p* < 0.01 by one-way ANOVA with Tukey’s post hoc test.

### SIRT1 improves bone regeneration in age-related osteoporosis by enhancing type H vessel density and bone formation through the Wnt/β-catenin signaling pathway

Micro-CT quantification at 2 weeks post-surgery revealed that SRT1720 significantly enhanced trabecular bone parameters in femoral defect areas, whereas EX527 impeded bone repair (Figures 7A and 7B). Histomorphometric evaluations through H&E and Masson staining corroborated these structural improvements, demonstrating enhanced collagen organization and mineralized matrix deposition in SRT1720-treated specimens (Figure 7A). Crucially, SRT1720 administration elevated type H vessel density compared with controls, with concurrent expansion of peri-vascular RUNX2+/ALP+ osteoprogenitor cell pools (Figures 7D, 7F, and 7H). Upregulated osteopontin (OPN) and HIF1α expression further supported these findings, counteracting age-associated regenerative impairment (Figures 7E, 7G, and 7H).

**Figure 7:**
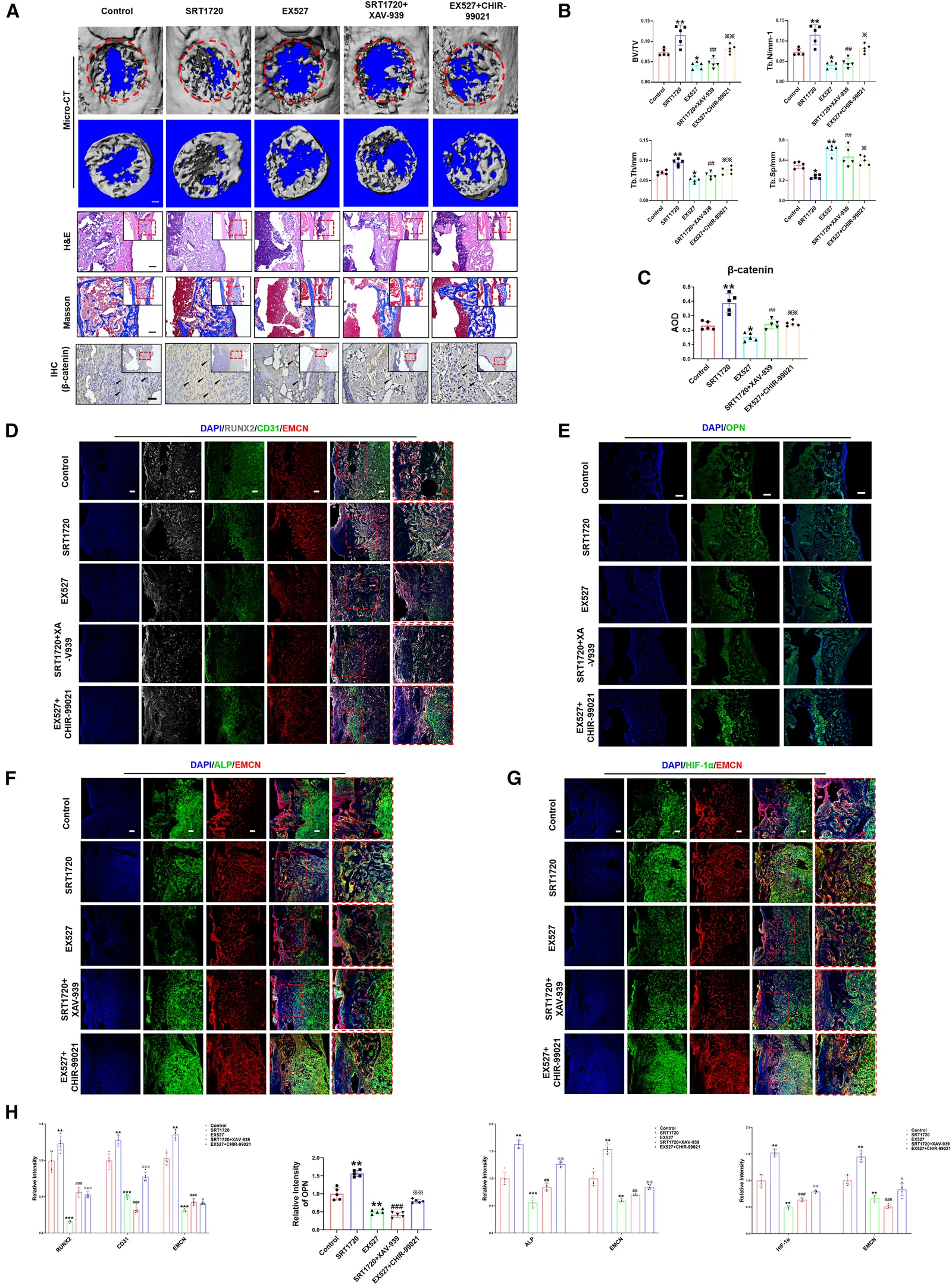
SIRT1 improves bone regeneration in age-related osteoporosis by enhancing type H vessel density and bone formation through the Wnt/β-catenin signaling pathway (A) Representative micro-CT reconstructions and histological characterization (H&E, Masson, and β-catenin immunohistochemical staining) of femoral defects at 2 weeks post-surgery. Red circles/rectangles demarcate the bone defect region. Scale bars: 200 μm (white), 100 μm (black). (B) Quantification of new bone mass within the defect area. (C) Immunohistochemical-based quantification of β-catenin-positive cells in the peri-defect region. (D) IF co-staining of type H vessels (CD31^hi^EMCN^hi^) and associated RUNX2+ osteoprogenitor cells at the defect site. Scale bars: 100 μm (white), 50 μm (red). (E–G) IF staining of osteogenic markers (OPN, E; ALP, F) and hypoxia-inducible factor 1α (HIF-1α, G) in the defect microenvironment. Scale bars: 100 μm (white), 50 μm (red). (H) Relative immunofluorescence quantification of markers shown in (D)–(G). Data are expressed as relative mean fluorescence intensity of positive area. Data presented as mean ± SD (*n* = 5 mice per group); ^∗^*p* < 0.05, ^∗∗^*p* < 0.01, ^∗∗∗^*p* < 0.001 (versus Control); ##*p* < 0.01, ###*p* < 0.001 (versus SRT1720); ※*p* < 0.05, ※※*p* < 0.01, ※※※*p* < 0.001 (versus EX527) by one-way ANOVA with Tukey’s post hoc test.

To establish Wnt/β-catenin signaling as the mechanistic cornerstone, pharmacological interventions were performed using CHIR-99021 (Wnt activator) and XAV-939 (Wnt inhibitor). CHIR-99021 partially rescued EX527-induced suppression of osteogenesis and angiogenesis, while XAV-939 attenuated SRT1720-mediated bone formation (Figures 7A, 7B, and 7D–7H). Immunohistochemical analysis confirmed β-catenin expression dynamics paralleled these functional outcomes (Figures 7A and 7C).

As shown in Figure S6, the inter-group differences in bone defect appearance at 1-week post-administration were consistent with those at 2 weeks. The regenerative paradigm was also replicated in mandibular defects, where SIRT1 activation similarly enhanced type H vessel density and osteoprogenitor cell recruitment, driving robust new bone formation (Figure S7). Wnt modulators exhibited consistent directional effects across skeletal sites, with CHIR-99021 and XAV-939 respectively rescuing or blunting SIRT1-mediated regeneration. Systemic safety assessments revealed no histopathological abnormalities in major organs (Figure S8).

## Discussion

Aging disrupts the coordinated relationship between angiogenesis and osteogenesis, contributing to osteoporosis and impaired bone regeneration (Chandra and Rajawat, 2021; Deng et al., 2021). In this study, we investigated whether SIRT1 contributes to age-related deterioration of type H vessels and osteogenic function. Using aged murine models, we observed that reduced SIRT1 expression correlates with decreased type H (CD31^hi^EMCN^hi^) vessel density and impaired osteogenic differentiation in both femoral and mandibular sites. Mechanistically, SIRT1 activates canonical Wnt/β-catenin signaling via β-catenin deacetylation and nuclear translocation. Pharmacological SIRT1 activation with SRT1720 improved type H vessel density and bone formation in aged mice and accelerated healing in femoral and mandibular defects. These findings suggest that targeting SIRT1 may offer a dual therapeutic strategy for age-related osteoporotic bone repair.

SIRT1 activation enhances reciprocal paracrine effects between aged BMSCs and endothelial cells *in vitro*. To explore the functional relationship between these two lineages, we employed bidirectional conditional media assays using senescent BMSCs and HUVECs. We observed that SIRT1-activated BMSCs secrete SLIT3, which promoted endothelial tube formation. Conversely, SIRT1-enhanced HUVECs produced pro-osteogenic factors including TGF-β, which augmented BMSC osteogenic differentiation. These reciprocal paracrine effects were consistently detected *in vitro*. However, whether such bidirectional paracrine relationships operate equivalently *in vivo* remains to be determined, as the complex bone microenvironment may involve additional cell types and direct cell-cell contacts not captured by these assays.

Our immunostaining confirmed that SIRT1 is expressed in endothelial cells of type H vessels across femoral and tibial compartments (Figure S9). Importantly, we observed that this expression is not strictly endothelial; SIRT1 is also concurrently expressed in adjacent perivascular cells and osteoblasts, pointing to a coordinated, multicellular regulatory role within the osteogenic niche. Notably, SIRT1 levels declined with age in all three skeletal sites, correlating with trabecular bone loss trajectories and consistent with clinical reports linking reduced SIRT1 to osteoporotic bone loss (El-Haj et al., 2016; Kumar et al., 2014). Beyond local deficits in type H vessels, broader system-wide vascular deterioration—including generalized endothelial senescence and impaired hemodynamics—likely exacerbates hypoxia and nutrient deprivation in the bone microenvironment (Prisby et al., 2007; Ramasamy et al., 2016). These global changes act synergistically with the breakdown of type H-mediated osteogenic support to drive skeletal degeneration in aging cohorts (Peng et al., 2020). Consequently, future studies targeting both specific vessel subtypes and the overall vascular microenvironment may offer more comprehensive therapeutic strategies.

In our experimental systems, β-catenin acts as a downstream mediator for SIRT1-enhanced osteogenic and endothelial functions. Our biochemical evidence indicates a direct SIRT1-β-catenin interaction and SIRT1-dependent β-catenin deacetylation, which correlates with increased β-catenin protein levels and enhanced nuclear translocation following SRT1720 treatment, leading to the upregulation of downstream Wnt targets. This aligns with previous findings that deacetylation protects β-catenin from proteasomal degradation (Chen et al., 2019; Guo et al., 2022; Xu et al., 2026; Yu et al., 2019). Functionally, knocking down *Ctnnb1* with three independent siRNAs consistently attenuated SIRT1-induced osteogenic and angiogenic capacities in senescent cells, accompanied by reduced RUNX2 and HIF1α expression. (Figure S10). This cross-siRNA concordance mitigates off-target concerns, although a genetic rescue experiment remains a limitation. *In vivo* pharmacological validation further supported this mechanism: a Wnt inhibitor reduced type H vessel density and bone formation in aged mice, while a Wnt activator enhanced these parameters. Finally, while SIRT1 can activate alternative pathways (e.g., PI3K/AKT/FOXO1) in different contexts (Liu et al., 2024), future studies using mass spectrometry are required to pinpoint the precise lysine residues deacetylated by SIRT1 and to elucidate its context-dependent pathway switching.

Several limitations should be acknowledged. First, this study focused specifically on type H vessels; whether SIRT1 selectively regulates this subtype or broadly affects the entire bone vasculature remains unclear. Second, our conditional medium approach does not capture direct cell-cell interactions or secretion dynamics. Third, all experiments used male mice, limiting generalizability to female physiology. Fourth, bone regeneration was assessed without biomechanical testing. Fifth, although our *in vitro* and *in vivo* pharmacological data demonstrate that SIRT1 activation enhances both endothelial and osteogenic functions, the current study does not establish a definitive *in vivo* mechanism regarding lineage coordination. The systemic nature of SRT1720 treatment precludes identifying a single cell-type-specific driver. Future studies using endothelial- or osteoblast-specific SIRT1 knockout or overexpression models are required to dissect cell-autonomous versus non-cell-autonomous contributions. Finally, while our *in vitro* HUVEC system effectively mimics stress-induced endothelial impairment, it does not fully replicate the complexity of true chronological aging. Consequently, our definitive conclusions regarding endothelial aging and type H vessel deterioration rely primarily on our *in vivo* data derived from naturally aged mice.

In summary, SIRT1 enhances both endothelial and osteogenic processes in the aged bone microenvironment, with β-catenin serving as a downstream mediator in these experimental systems. Enhancing SIRT1 function improves type H vessel density and bone formation in aged mice and accelerates femoral and mandibular defect regeneration. These findings suggest that targeting SIRT1 may improve age-related bone regeneration by enhancing both lineages, offering a dual therapeutic strategy for geriatric osteoporotic bone repair.

## Resource availability

### Lead contact

Requests for further information and resources should be directed to and will be fulfilled by the lead contact, En Luo (luoen521125@sina.com).

### Materials availability

All reagents generated in this study are available upon request to the lead contact.

### Data and code availability

The datasets for RNA-seq were obtained from GSE163515. All other raw data and codes are available upon request to the lead contact.

## Acknowledgments

The authors would like to thank Yinghui Wen (State Key Laboratory of Oral Diseases, West China Hospital of Stomatology, Sichuan University) for help in cell culture. This study was supported by the 10.13039/501100001809National Natural Science Foundation of China (grant/award no. 82370932); Sichuan Provincial Department of Science and Technology Fund Project (grant/award nos. 2024YFFK0204 and 2024NSFSC1588); Research and Develop Program, West China Hospital of Stomatology, 10.13039/501100004912Sichuan University (grant/award no. RD-02-202506); 10.13039/501100002858China Postdoctoral Science Foundation (grant/award no. 2024M752269); and Special Funding of the Sichuan Province Postdoctoral Science Fund (grant/award no. TB2024082).

## Author contributions

S.L., conceptualization, investigation, methodology, visualization, formal analysis, and writing – original draft preparation; Q.Y., investigation, methodology, data collection, and writing – review and editing; H.L., investigation and methodology; J.S., investigation and methodology; Y.L., writing – review and editing; Z.Z., writing – review and editing; E.L., conceptualization, supervision, funding acquisition, and writing – review and editing.

## Declaration of interests

The authors declare no competing interests.

## STAR★Methods

### Key resources table

**Table:** 

REAGENT or RESOURCE	SOURCE	IDENTIFIER
**Antibodies**		

Anti-αTubulin Antibody	HUABIO	Cat#ER130905; RRID: AB_3069023
Anti-ALP Antibody	HUABIO	Cat#ET1601-21; RRID: AB_3069604
Anti-RUNX2 Antibody (mouse)	Santa Cruz	Cat#sc-101145; AB_1128251
Anti-RUNX2 Antibody (Rabbit)	HUABIO	Cat#ET1612-47; RRID: AB_2924311
Anti-CD31 Antibody	HUABIO	Cat#ER31219; RRID: AB_3069567
Anti-EMCN Antibody	Santa Cruz	Cat#sc-65495; RRID:AB_2100037
Anti-HIF1α Antibody	Affinity	Cat#AF1009; RRID:AB_2835328
Anti-H3 Antibody	HUABIO	Cat#EM30605; RRID:AB_2890027
Anti-Active β-Catenin Antibody	abcam	Cat#ab246504
Anti-CyclinD1 Antibody	Proteintech	Cat#26939-1-AP; RRID:AB_2880691
Anti-LEF-1 Antibody	HUABIO	Cat#HA500273; RRID: AB_3071369
Anti-β-Catenin Antibody (mouse)	HUABIO	Cat#EM0306; RRID:AB_3068732
Anti-β-Catenin Antibody (rabbit)	HUABIO	Cat#ET1601-5; RRID:AB_3069616
Anti-IgG Antibody	Cell Signaling Technology	Cat#2729; RRID:AB_1031062
Anti-SIRT1 Antibody (mouse)	Santa Cruz	Cat#sc-74465; RRID:AB_1129462
Anti-SIRT1 Antibody (rabbit)	HUABIO	Cat#ET1603-3; RRID:AB_3069678
Anti-Acetyl-lysin Antibody	Santa Cruz	Cat#sc-32268; RRID:AB_627898
Anti-P16 Antibody	abcam	Cat#ab51243; RRID:AB_2059963
Anti-P53 Antibody	abcam	Cat#ab131442; RRID: AB_11155283
Anti-PDGFβ Antibody	HUABIO	Cat#ET1605-20; RRID:AB_3069707
iFluor™ 488 Conjugated Goat anti-mouse IgG polyclonal Antibody	HUABIO	Cat#HA1125; RRID:AB_3740920
iFluor™ 488 Conjugated Goat anti-rabbit IgG polyclonal Antibody	HUABIO	Cat#HA1121; RRID:AB_3675804
iFluor™ 647 Conjugated Goat anti-mouse IgG polyclonal Antibody	HUABIO	Cat#HA1127
HRP Conjugated Goat anti-Rabbit IgG polyclonal Antibody	HUABIO	Cat#HA1001; RRID:AB_2819166
HRP Conjugated Goat anti-Mouse IgG polyclonal Antibody	HUABIO	Cat#HA1006; RRID:AB_2819167
Dylight 549, Goat Anti-Rat IgG	Abbkine	Cat#A23340

**Biological samples**		

Primary bone marrow mesenchymal stem cells	Isolated from mouse bone marrow in this study	N/A

**Chemicals, peptides, and recombinant proteins**		

H_2_O_2_ solution	Sigma Aldrich	Cat#H1009
Tissue-Tek® OCT compound	Sakura Finetek	Cat#4583
4% paraformaldehyde	Biosharp	Cat# BL539A
Triton X-100	Biofroxx	Cat# 1139ML100
Goat Serum	Solarbio	Cat#SL038
EDTA Antigen Retrieval Solution (50X)	Solarbio	Cat#C1034
TRIzol reagent	Invitrogen	Cat#15596026
RIPA Buffer	Solarbio	Cat#R0010
β glycerophosphate pentahydrate	Sigma Aldrich	Cat#SLCF2153
Ascorbic acid	Merck	Cat#A4544
Dexamethasone	Solarbio	Cat#6090
SRT1720	APEXBIO	Cat#A4180
EX527	APEXBIO	Cat#A4181
Alizarin Red S Solution	Solarbio	Cat# G1450
Crystal Violet Ammonium Oxalate Solution	Solarbio	Cat#G1063
Matrigel	Corning	Cat#354230
TRITC Phalloidin	Solarbio	Cat#CA1610
EndoFectin™ transfection reagent	GeneCopoeia	Cat#EF013
Serum free Opti MEM™ reduced serum medium	Gibco	Cat#31985062
IP lysis buffer	Beyotime	Cat#P0013
PMSF	Sangon	Cat#A610560
Phosphatase inhibitors	Sangon	Cat#C600007
Protein A/G magnetic beads	Bimake	Cat# B23202
Ketamine	Fujian Gutian Pharmaceutical	N/A
Xylazine	Sigma-Aldrich	Cat#X1251

**Critical commercial assays**		

Senescence-Associated β-Galactosidase (SA-β-Gal) Stain Kit	Solarbio	Cat# G1580
Hematoxylin-Eosin (H&E) Stain Kit	Solarbio	Cat# G1120
Masson’s Trichrome Stain Kit	Solarbio	Cat# G1340
DAB Substrate Kit	Solarbio	Cat# DA1010
PrimeScript FAST RT reagent Kit with gDNA Eraser	Takara	Cat# RR092S
SYBR Green 2× Master Mix	Selleck	Cat#B21702
Nucleoprotein Extraction Kit	Sangon Biotech	Cat#C510001
BCIP/NBT Alkaline Phosphatase Color Development Kit	Beyotime	Cat#C3206
Mouse SLIT3 Elisa Kit	MEIMIAN	Cat#MM-46732M2
Human TGFβ Elisa Kit	MEIMIAN	Cat#MM-46732M2
Treble-Fluorescence immunohistochemical mouse/rabbit kit	Immunoway	Cat#RS0035

**Deposited data**		

RNA-seq data	Rodrigues et al. (Rodrigues et al., 2022)	GEO: GSE163515

**Experimental models: Cell lines**		

Human Umbilical Vein Endothelial Cells	Cell Bank of the Chinese Academy of Sciences	SCSP-5536

**Experimental models: Organisms/strains**		

C57BL/6 mice	West China Medical Laboratory Animal Center of Sichuan University	N/A

**Oligonucleotides**		

Mouse *Gapdh* Forward: AGGTCGGTGTGAACGGATTTG	This Paper	N/A
Mouse *Gapdh* Reverse: TGTAGACCATGTAGTTGAGGTCA	This Paper	N/A
Mouse *Runx2* Forward: TTCAACGATCTGAGATTTGTGGG	This Paper	N/A
Mouse *Runx2* Reverse: GGATGAGGAATGCGCCCTA	This Paper	N/A
Mouse *Alp* Forward: CCAACTCTTTTGTGCCAGAGA	This Paper	N/A
Mouse *Alp* Reverse: GGCTACATTGGTGTTGAGCTTTT	This Paper	N/A
Mouse *P53* Forward: CTCTCCCCCGCAAAAGAAAAA	This Paper	N/A
Mouse *P53* Reverse: CGGAACATCTCGAAGCGTTTA	This Paper	N/A
Mouse *P21* Forward: CCTGGTGATGTCCGACCTG	This Paper	N/A
Mouse *P21* Reverse: CCATGAGCGCATCGCAATC	This Paper	N/A
Mouse *P16* Forward: CGCAGGTTCTTGGTCACTGT	This Paper	N/A
Mouse *P16* Reverse: TGTTCACGAAAGCCAGAGCG	This Paper	N/A
Homo *Gapdh* Forward: GGAGCGAGATCCCTCCAAAAT	This Paper	N/A
Homo *Gapdh* Reverse: GGCTGTTGTCATACTTCTCATGG	This Paper	N/A
Homo *P53* Forward: GAGGTTGGCTCTGACTGTACC	This Paper	N/A
Homo *P53* Reverse: TCCGTCCCAGTAGATTACCAC	This Paper	N/A
Homo *P16* Forward: ATGGAGCCTTCGGCTGACT	This Paper	N/A
Homo *P16* Reverse: GTAACTATTCGGTGCGTTGGG	This Paper	N/A
Homo *P21* Forward: TGTCCGTCAGAACCCATGC	This Paper	N/A
Homo *P21* Reverse: AAAGTCGAAGTTCCATCGCTC	This Paper	N/A
Primer for *Ctnnb1,* see Table S1	This Paper	N/A
siRNA for *Ctnnb1,* see Table S1	This Paper	N/A

**Software and algorithms**		

Image J	Schneider et al. (Schneider et al., 2012)	https://imagej.net/ij/
R software version 4.2.0	Yu et al. (Yu et al., 2012)	https://www.r-project.org/
IBM SPSS Statistics software version 29.0	Kileo et al. (Kileo et al., 2026)	https://www.ibm.com/products/spss-statistics

### Experimental model and study participant details

#### Animals

Male C57BL/6 mice at three distinct age groups (2-month-old, 8-month-old, and 18-month-old, *n* = 5 per group) were obtained from the West China Medical Laboratory Animal Center of Sichuan University. Mice were housed under specific pathogen-free (SPF) conditions with a strict 12-h light/dark cycle (lights on at 7:00 a.m.), temperature of 22 ± 2°C, and relative humidity of 50 ± 10%, with *ad libitum* access to standard laboratory chow and water. Animals were group-housed (up to five per cage) in individually ventilated cages (IVCs) with enriched bedding. All mice were drug-naïve and had not undergone any prior experimental procedures. For tissue collection, animals were euthanized by cervical dislocation under isoflurane anesthesia, consistent with AVMA guidelines for rapid physical methods of euthanasia. Mice of the same age were randomly assigned to experimental groups. The experimental protocol was reviewed and approved by the Ethics Committee of West China Hospital of Stomatology, Sichuan University (Approval No. WCHSIRB-D-2022-071), and all animal experiments were performed in accordance with the NIH Guide for the Care and Use of Laboratory Animals (8th edition) and the ARRIVE guidelines 2.0.

#### Cell lines and primary cell cultures

Human umbilical vein endothelial cells (HUVECs) were maintained in Dulbecco's Modified Eagle Medium (DMEM, Gibco, USA) supplemented with 10% fetal bovine serum (FBS, Gibco, USA) and 1% penicillin/streptomycin (Gibco, USA). Bone marrow-derived mesenchymal stem cells (BMSCs) were isolated from the femurs and tibiae of 3-week-old male C57BL/6 mice via marrow flushing using α-Minimum Essential Medium (α-MEM, Gibco, USA) containing 10% FBS. BMSCs at passage 3 (P3) were used for all experiments. Both cell types were cultured under standard conditions (37°C, 5% CO_2_, 95% humidity), with medium changes every 2–3 days. Commercially sourced HUVECs were derived from a mixed-sex donor pool, a limitation we acknowledge regarding sex as a biological variable. Primary BMSCs were derived from male mice. While STR profiling was not performed, BMSC identity was confirmed via morphological observation and osteogenic/adipogenic differentiation assays.

### Method details

#### Induction of cellular senescence

Cellular senescence was induced via two distinct approaches. For oxidative stress-induced senescence, cells were treated with 200 μM hydrogen peroxide (H_2_O_2_, HP1009, Sigma-Aldrich, USA) in complete culture medium for 24 h. For replicative senescence, BMSCs were serially passaged until passage 8 (P8), at which point cells exhibited replicative exhaustion as previously described (Kretlow et al., 2008; Xu et al., 2020). Senescence induction efficiency was confirmed by senescence-associated β-galactosidase (SA-β-gal) staining (G1580, Solarbio, China) and by analyzing the expression of senescence markers p53, p21, and p16 via western blot and quantitative real-time PCR. All experiments were performed with at least three independent replicates.

#### Micro-computed tomography (micro-CT) analysis

Bone specimens were systematically harvested and subjected to micro-CT analysis (Scanco Medical, Switzerland) with standardized scanning parameters (10 μm scanning resolution, 70 kV voltage, 114 μA current). For long bone morphometry, femoral and tibial regions of interest (ROIs) were defined as a 2 mm trabecular compartment (200 consecutive slices) extending distally from the growth plate-metaphyseal junction. For jaw specimens, three-dimensional reconstructions focused on the periapical region of the first molar root apex. Quantitative morphometric evaluation included four key trabecular parameters: bone volume/total volume (BV/TV), trabecular number (Tb.N), trabecular thickness (Tb.Th), and trabecular separation (Tb.Sp), calculated using manufacturer-certified analysis software. Five animals per group were analyzed, with three ROIs measured per animal.

#### Histological staining (H&E, Masson, and SA-β-gal)

Decalcified bone specimens were processed through graded ethanol dehydration, embedded in paraffin, and sectioned into 4 μm slices for H&E (G1120, Solarbio, China) and Masson staining (G1340, Solarbio, China). For SA-β-gal detection, tissues were embedded in Tissue-Tek® OCT compound (4583, Sakura Finetek, Japan), cryosectioned into 8 μm slices at -20°C, and stained using a SA-β-gal staining kit (G1580, Solarbio, China) following the manufacturer's protocol. For *in vitro* SA-β-gal staining, fixed cells were stained in parallel with DAPI nuclear counterstaining. All samples were imaged under standardized conditions using an optical microscope (Leica, Germany). Five random fields of view were selected per section for quantitative analysis.

#### Immunofluorescence (IF) and immunohistochemistry (IHC)

For cultured cells, cells were fixed with 4% paraformaldehyde (BL539A; Biosharp, China) for 15 min, permeabilized with 0.5% Triton X-100 (1139ML100, Biofroxx, Germany) for 10 min, and blocked with 10% goat serum (SL038, Solarbio, China) for 1 h at room temperature. Primary antibodies were incubated overnight at 4°C, followed by incubation with fluorophore-conjugated secondary antibodies for 1 h at room temperature and DAPI nuclear staining. For tissue IF, frozen sections (30 μm for type H vessel analysis) underwent blocking and antibody staining without antigen retrieval, whereas paraffin sections were subjected to antigen retrieval in EDTA buffer (C1034, Solarbio, China) prior to staining. For IHC, paraffin sections underwent antigen retrieval, blocking with 10% goat serum, and sequential incubations with primary antibodies (overnight at 4°C) and HRP-conjugated secondary antibodies (1 h at room temperature). Signals were visualized using DAB substrate (DA1010, Solarbio, China), and nuclei were counterstained with hematoxylin. Negative controls (omission of primary antibody) were included in all staining runs.

#### RNA extraction, quantitative real-time PCR (qRT-PCR), and western blot

Total RNA was isolated from cultured cells using TRIzol reagent (15596026, Invitrogen, USA) according to the manufacturer's instructions. Complementary DNA (cDNA) synthesis was performed using the PrimeScript™ RT Reagent Kit with gDNA Eraser (RR092S, Takara, Japan). qRT-PCR was carried out using SYBR Green 2× Master Mix (B21702, Selleck, USA) on a CFX96 Real-Time PCR Detection System (Applied Biosystems, USA). Relative gene expression was calculated using the 2^-ΔΔCt^ method, with *Gapdh* as the internal control. Each sample was run in triplicate, and at least three independent biological replicates were performed.

For protein analysis, whole-cell lysates were prepared using RIPA lysis buffer (R0010, Solarbio, China). Nuclear fractions were isolated using a commercial nuclear extraction kit (C510001, Sangon Biotech, China) following the recommended protocol. Protein samples were separated by SDS-PAGE and subsequently transferred to PVDF membranes (0000204510, Millipore, USA). After blocking with 5% non-fat milk in TBST for 1 h at room temperature, membranes were incubated overnight at 4°C with primary antibodies, followed by 1 h incubation with HRP-conjugated secondary antibodies at room temperature. Protein signals were detected by ECL and visualized using a ChemiDoc Imaging System (Bio-Rad, USA). Band intensities were quantified using Image J software (NIH, USA) (Sen et al., 2010) and normalized to loading controls (GAPDH, β-Actin or Histone H3). Antibody details are provided in Table S2.

#### Osteogenic differentiation and staining (ALP and ARS)

BMSCs were cultured in osteogenic induction medium composed of complete α-MEM supplemented with 10 mM β-glycerophosphate pentahydrate (SLCF2153, Sigma Aldrich, USA), 10 mM ascorbic acid (A4544, Merck, Germany), and 1 mM dexamethasone (ID6090, Solarbio, China). Pharmacological modulation was performed using SRT1720 (SIRT1 activator, A4180, APEXBIO, USA) at concentrations of 0, 0.5, 1.0, and 2.5 μM, and EX527 (SIRT1 inhibitor, A4181, APEXBIO, USA) at concentrations of 0, 2.5, 5.0, and 10.0 μM, in a concentration-dependent design. To assess osteogenic differentiation progression, alkaline phosphatase (ALP) activity was evaluated on day 7 post-induction using an ALP staining kit (C3206, Beyotime, China), followed by Alizarin Red S (ARS, G1452, Solarbio, China) mineralization assessment on day 14. For quantification, stained cultures were evaluated using Image J software.

#### Cell migration and angiogenic potential assessment

Wound healing assay: Confluent cell monolayers were scratched using a sterile 200 μL pipette tip. After gentle washing to remove detached cells, serum-free basal medium was applied. Wound closure was monitored by microscopy (Olympus, Japan) at baseline (0 h) and 24 h post-scratch. Wound areas were quantified using Image J software, and the percentage of wound closure was calculated relative to baseline. Experiments were performed in triplicate wells per condition.

Transwell migration assay: Cell suspensions (2 × 10^4^ cells per well in serum-free medium) were loaded into the upper chambers of 24-well Transwell plates (8 μm pore size, Corning, USA) containing modulators (SRT1720 or EX527). The lower chambers contained medium with 10% FBS as a chemoattractant or serum-free medium as a control. After 24 h incubation at 37°C, non-migrated cells on the upper surface were removed with a cotton swab, and transmigrated cells on the lower surface were fixed with 4% paraformaldehyde and stained with 0.1% crystal violet solution (G1063, Solarbio, China). Stained cells were counted in five random fields per well under a light microscope.

Tube formation assay: Matrigel (354230, Corning, USA) was polymerized in 48-well plates at 37°C for 30 min. HUVECs (2 × 10^4^ cells per well) were seeded on the Matrigel and cultured for 6 h. Following fixation with 4% paraformaldehyde, endothelial networks were visualized through F-actin staining (CA1610, Abcam, USA). Tubular structures were analyzed using the Angiogenesis Analyzer plugin of Image J software, measuring number of nodes, number of meshes and total branches length.

#### Paracrine interaction model establishment

Conditional medium (CM) was collected from BMSCs and HUVECs to establish a paracrine-mediated co-culture system. For BMSC-derived CM, BMSCs were cultured in α-MEM with 10% FBS for 48 h; the supernatant was collected, centrifuged at 3,000 rpm for 20 min, and applied to HUVECs. For HUVEC-derived CM, HUVECs were cultured in DMEM with 10% FBS for 48 h; the supernatant was similarly collected and applied to BMSCs. Recipient cells were treated with CM for varying durations: 5 days for qRT-PCR and western blot analysis, or as required for wound healing, Transwell, and tube formation assays. Quantitative analysis of paracrine factors SLIT3 (MM-46732M2, MEIMIAN, China) and TGF-β (MM-0090H1, MEIMIAN, China) was performed using commercial ELISA kits following the manufacturer's protocol. Each condition was tested in triplicate.

#### siRNA transfection

Following 24 h cell attachment in antibiotic-free medium, *Ctnnb1* (encoding β-Catenin)-specific siRNAs were complexed with 5% (v/v) EndoFectin™ transfection reagent (EF013, GeneCopoeia, China) in serum-free Opti-MEM™ reduced serum medium (31985062, Gibco, USA). A non-targeting scrambled siRNA served as the negative control. Transfection complexes were incubated at room temperature for 20 min prior to addition to cells. Cells were transfected for 24 h, after which BMSCs and HUVECs underwent subsequent experimental treatments and functional analyses. Knockdown efficiency was validated by qRT-PCR and western blot.

#### Co-immunoprecipitation (Co-IP)

Cell lysates were prepared using IP lysis buffer (P0013, Beyotime, China) containing 1 mM PMSF (A610560, Sangon Biotech, China) and phosphatase inhibitors (C600007, Sangon Biotech, China). After centrifugation (12,000 × g, 15 min, 4°C), the supernatant was divided: one aliquot served as input control, and the remainder underwent pre-clearing with normal rabbit IgG and protein A/G magnetic beads (B23202, Bimake, USA) for 1 h at 4°C. The pre-cleared lysate was incubated overnight at 4°C with IgG control or anti-β-Catenin antibody, followed by 2 h incubation with fresh protein A/G beads. Beads were washed five times with IP lysis buffer, and bound proteins were eluted by boiling in 2× SDS loading buffer for immunoblotting analysis.

#### RNA-sequencing (RNA-seq) analysis

Transcriptomic profiling of bone endothelial cells isolated from 2-week-old and 65-week-old male mice was performed using the publicly available GEO dataset GSE163515 (Rodrigues et al., 2022). Differentially expressed genes (DEGs) were identified with stringent thresholds (|fold change| > 1.5, adjusted *p* < 0.05) through comparative analysis between age groups. Functional annotation of the DEGs was subsequently conducted via Gene Ontology (GO) enrichment analysis using the clusterProfiler package in R software (v4.2.0) (Yu et al., 2012).

#### Bone defect model and drug intervention *in vivo*

Under ketamine (100 mg/kg, Fujian Gutian Pharmaceutical, China) and xylazine (10 mg/kg, X1251, Sigma-Aldrich, USA) anesthesia, two standardized 1 mm bone defects were surgically created: a femoral defect located below the growth plate using a dental drill, and a mandibular defect at the distal masseter ridge via a submandibular approach. Mice were randomly divided into five groups (*n* = 10 per group): (1) Vehicle control (0.9% saline); (2) SRT1720 (25 mg/kg/day); (3) EX527 (2 mg/kg/day); (4) SRT1720 (25 mg/kg/day) + XAV939 (Wnt inhibitor, 2.5 mg/kg every 2 days); and (5) EX527 (2 mg/kg/day) + CHIR99021 (Wnt activator, 50 mg/kg every 2 days). All pharmacological agents were administered via intraperitoneal injection throughout the 14-day post-surgical intervention period. Investigators performing surgeries and subsequent analyses were blinded to treatment allocation. Mice were euthanized by cervical dislocation under isoflurane anesthesia at the endpoint. Bone regeneration was evaluated by micro-CT and histological analyses following specimen collection. Concurrent histopathological examination of myocardial, hepatic, and renal parenchyma was performed to monitor treatment-associated toxicity.

### Quantification and statistical analysis

All data are presented as mean ± standard deviation (SD) from at least three independent experiments unless otherwise indicated. Statistical analyses were performed using IBM SPSS Statistics (Version 20.0, Armonk, NY) (Kileo et al., 2026). Comparisons between two groups were analyzed using unpaired two-tailed Student's t-test. Comparisons among multiple groups were analyzed using one-way ANOVA followed by Tukey's post-hoc test. A *p*-value < 0.05 was considered statistically significant.

## Supplemental information

Document S1. Figures S1–S10 and Table S1

Document S2. Article plus supplemental information
