# Supplementary material for: SIRT1 promotes endothelial activity and osteogenesis in vitro and ameliorates age-related skeletal regeneration defects in vivo
Source: Stem Cell Reports. 2026 Sep 3;21(10):103063. doi: 10.1016/j.stemcr.2026.103063 (PMC13681407; doi:10.1016/j.stemcr.2026.103063)
Supplement: Supplementary file 1 [file mmc1.pdf]

Stem Cell Reports, Volume *21*

Supplemental Information

SIRT1 promotes endothelial activity and osteogenesis *in vitro* and ameliorates age-related skeletal regeneration defects *in vivo*

Shibo Liu, Jingjing Shao, Qiheng Yang, Hanghang Liu, Yao Liu, Zizhuo Zheng, and En Luo

Supplemental Figures

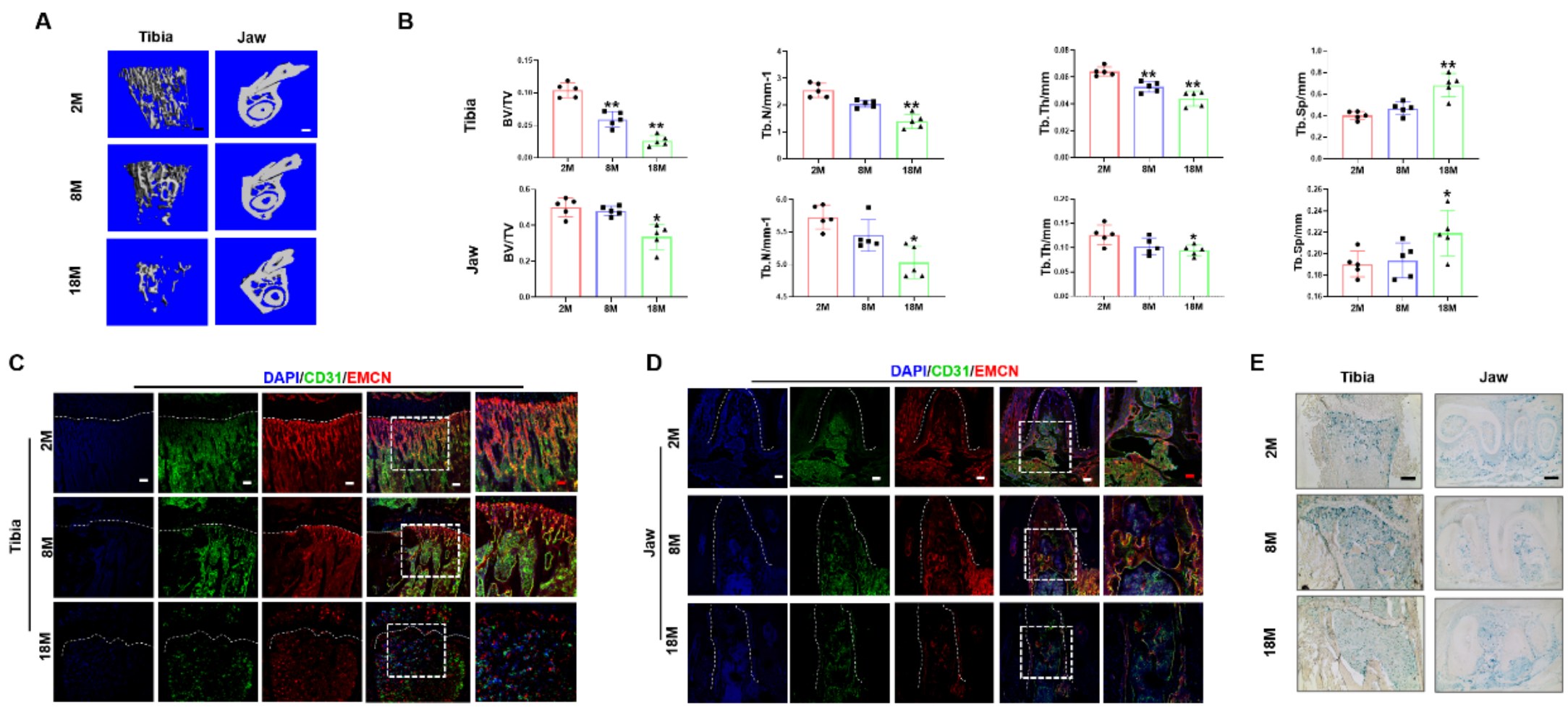

Figure S1 Age-related changes of type H vessels and bone in the tibia and mandible, related to Figure 1. (A, B) Micro-CT reconstructed images and corresponding quantitative analysis of tibia and mandible. Scale bars: 1 mm (white); 200 μm (black). (C, D) Immunofluorescence co-staining of $CD31^{hi}EMCN^{hi}$ - type H vessels in the tibia and mandible. Scale bar: 100 μm(white); 50 μm (red). (E) SA-β-gal staining in the tibia and mandible. Scale bar: 100 μm. Data presented as mean ± SD; **p* < 0.05, ***p* < 0.01 by one-way ANOVA with Tukey's post-hoc test.

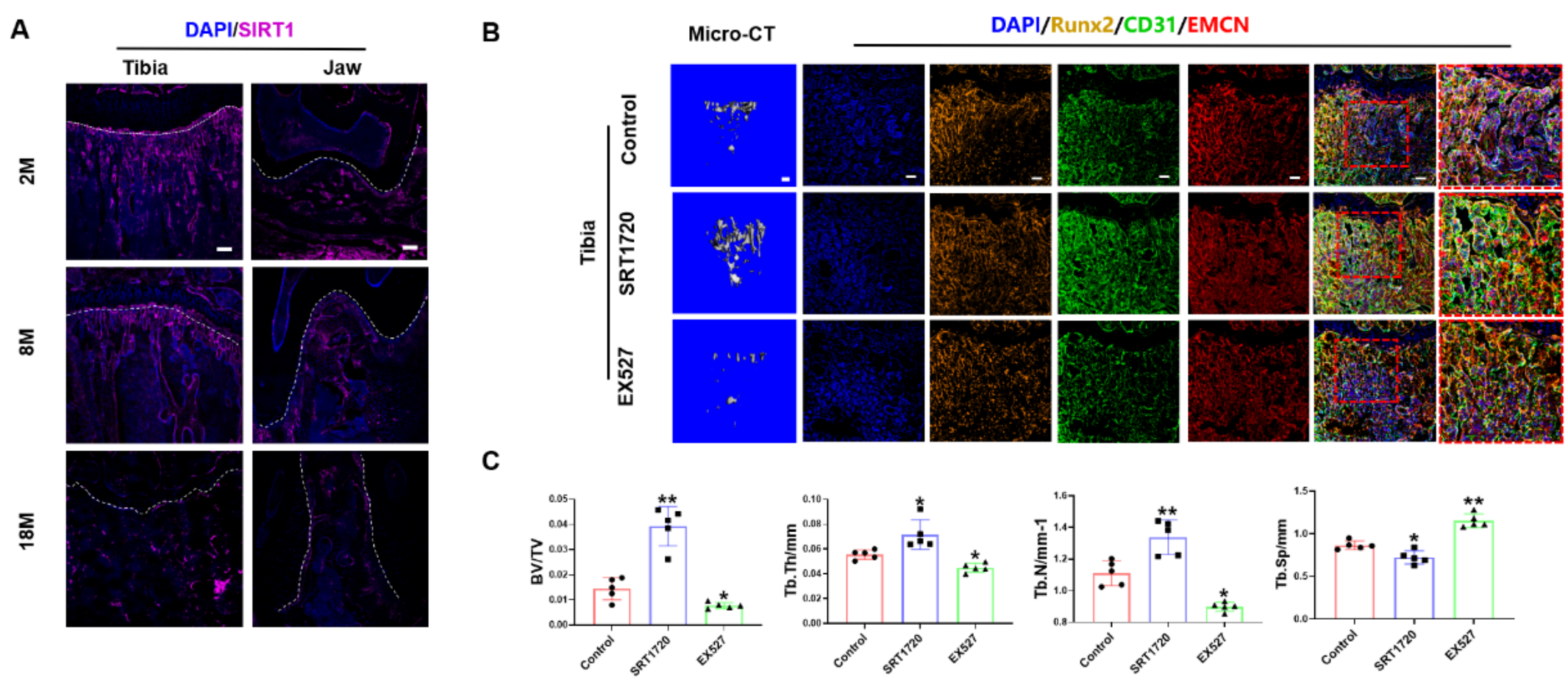

Figure S2 SIRT1 enhances type H vessel and osteogenic activity in the tibia, related to

Figure 1. (A) Expression of SIRT1 in the tibia and jaw across various age groups. Scale bar: 100 μm. (B-C) SIRT1 activation (SRT1720) and inhibition (EX527) bidirectionally altered type H vessel and osteogenic activity in the tibia, presented by Micro-CT assay and the Immunofluorescence staining. Scale bar: 100 μm(white); 50 μm (red). Data presented as mean ± SD; *$p < 0.05$, **$p < 0.01$ by one-way ANOVA with Tukey's post-hoc test.

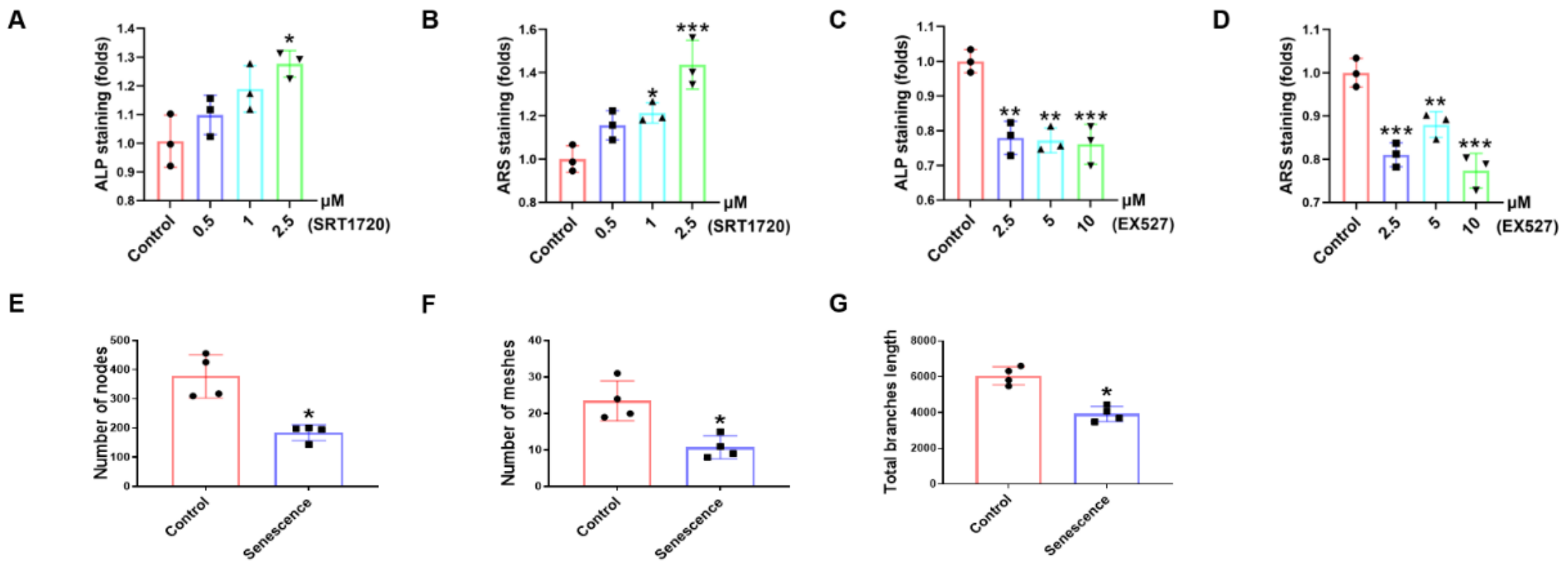

Figure S3 Complementary quantification of staining results, related to Figure 2. (A-D) ALP and ARS staining in senescent BMSCs. (E-F) Tube formation assay in HUVECs. Scale bar: 200 μm. Data presented as mean ± SD; *$p < 0.05$, **$p < 0.01$, and ***$p < 0.001$ by one-way ANOVA with Tukey's post-hoc test and unpaired two tailed Student's t test. Alkaline phosphatase: ALP; Alizarin Red S: ARS; Bone marrow stromal cells: BMSCs.

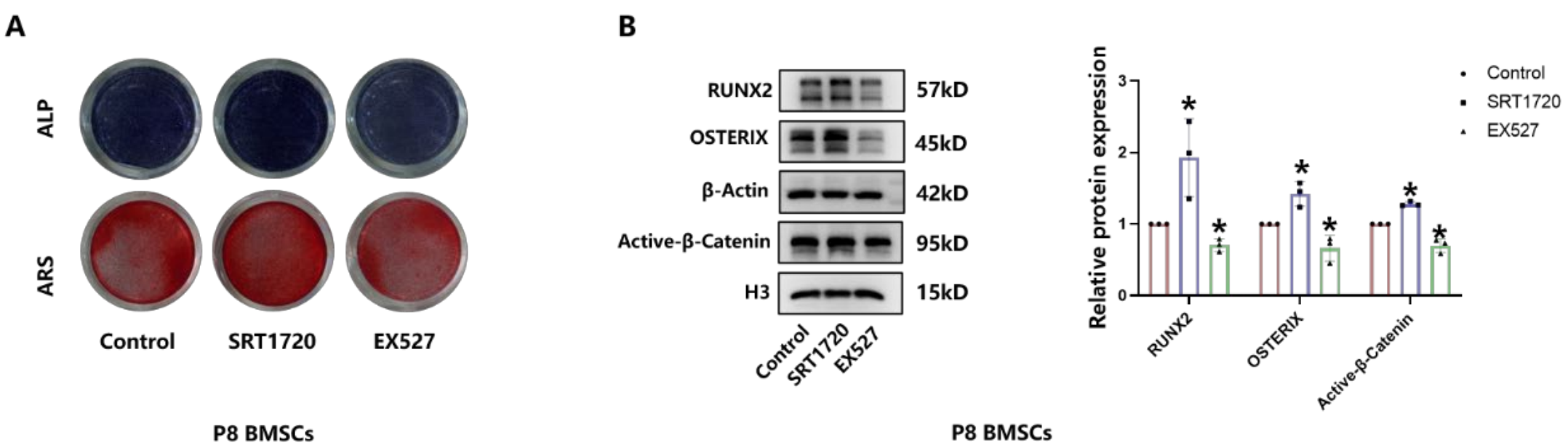

Figure S4 SIRT1 preserves the osteogenic capacity of replicatively senescent BMSCs, related to Figure 2. A, ALP and ARS staining in P8 BMSCs; B, Western blot analysis for p8 BMSCs. P8, BMSCs were continuously passaged until passage 8. Data presented as mean ±

SD; *$p < 0.05$ by one-way ANOVA with Tukey's post-hoc test.

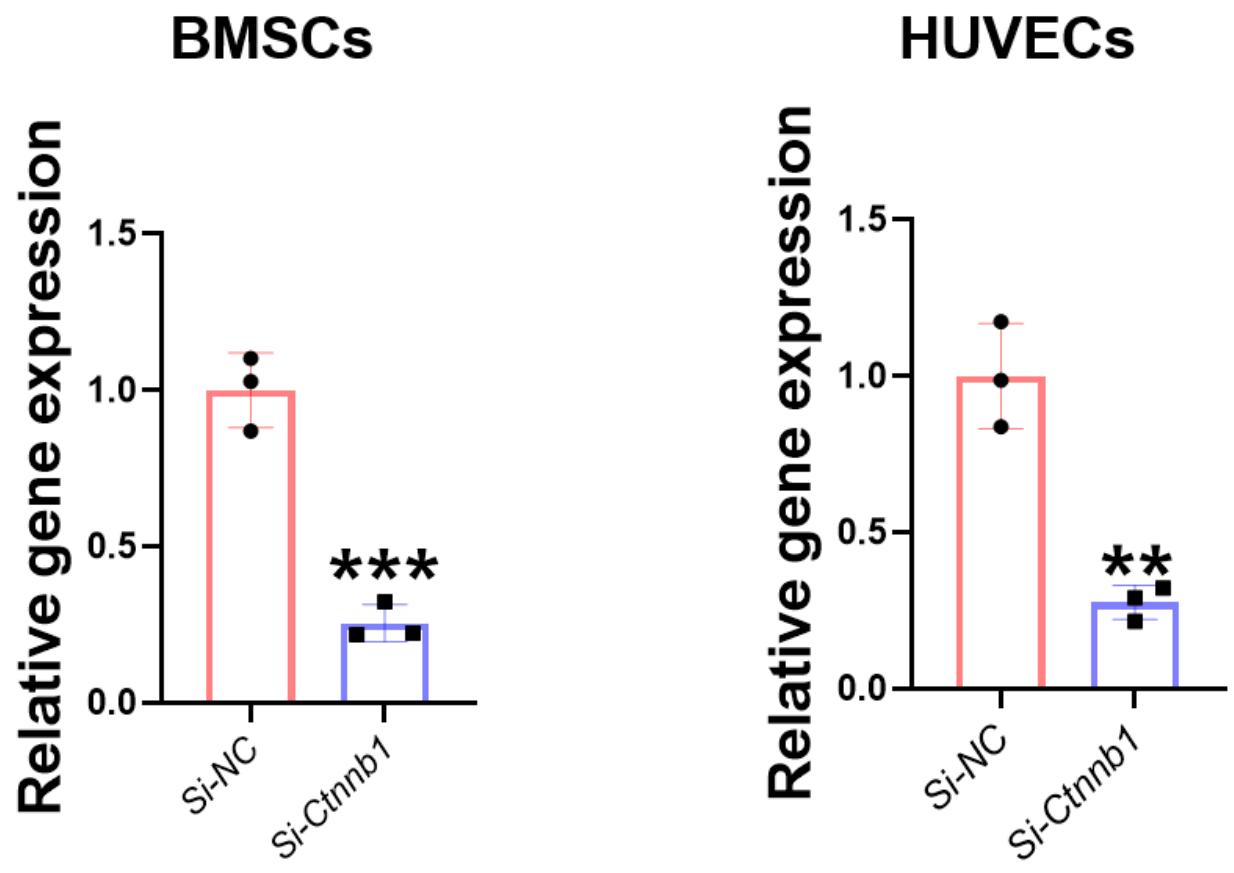

Figure S5 Validation of β-catenin knockdown efficiency in senescent BMSCs and HUVECs, related to Figure 5. Data presented as mean ± SD; **$p < 0.01$, ***$p < 0.001$ by unpaired two tailed Student's t test.

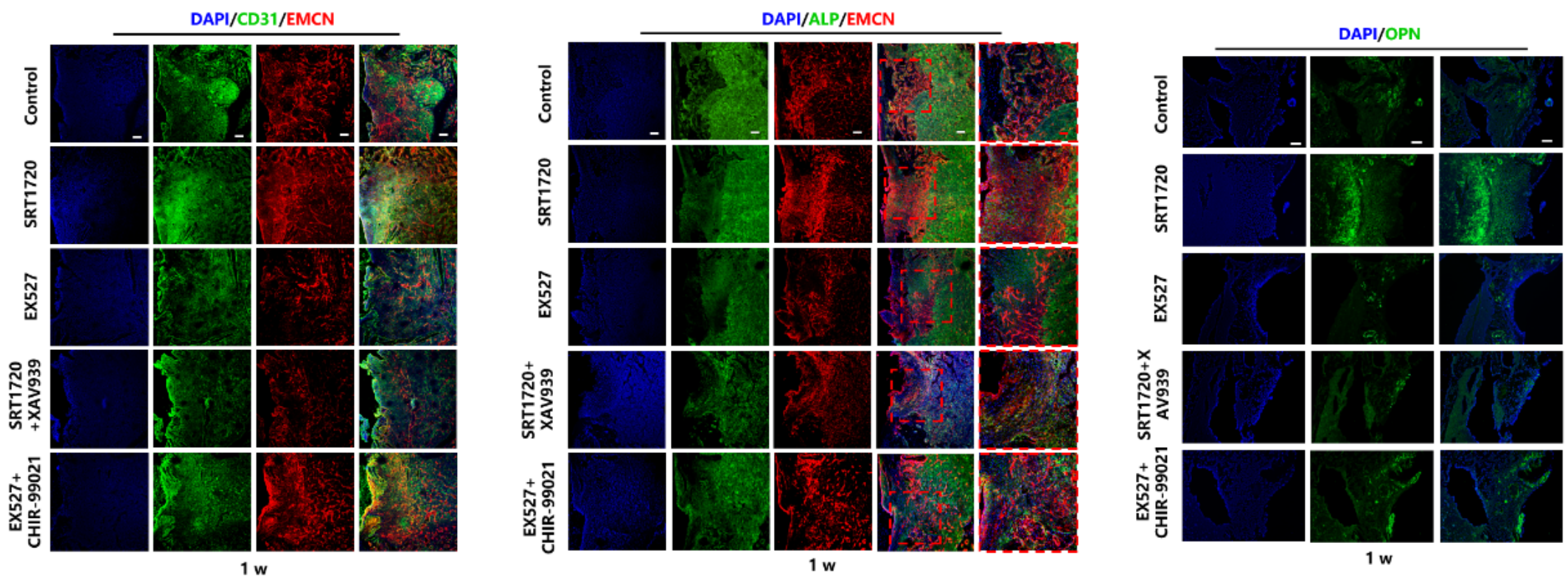

Figure S6 Bone defect status in aged mice at one-week post-administration, related to Figure 7. Scale bar: 100μm (white); 50μm (red).

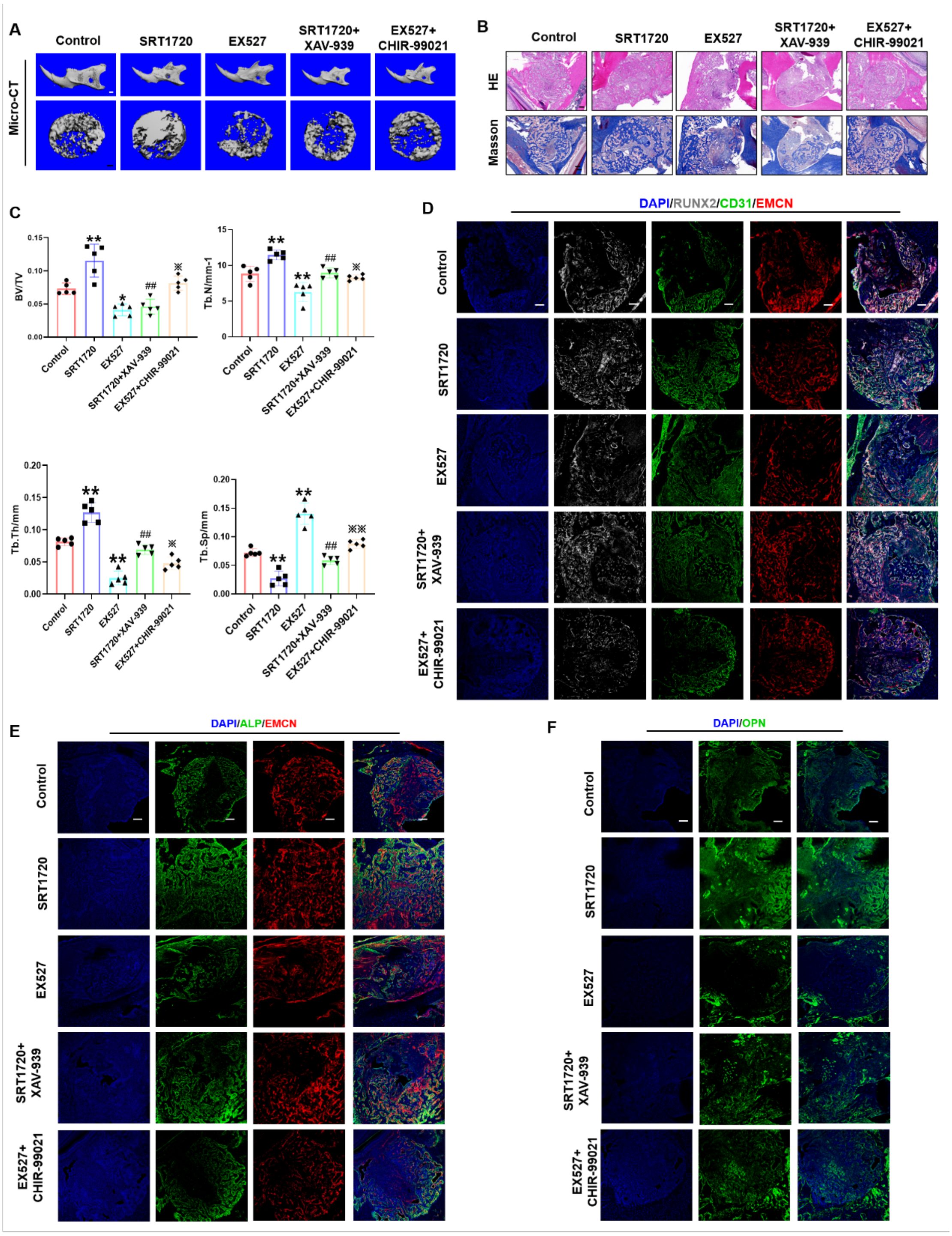

Figure S7 SIRT1-enhanced vascularized osteogenesis in aged jaw bone defects via Wnt/β-Catenin signaling, related to Figure 7. (A, B) Jaw defect regeneration assessed by micro-CT reconstruction (A) and histopathological evaluation (B). Scale bars: 1 mm (white), 100 μm (black). (C) Quantified osteogenic efficacy within the jaw defect zone. (D) Immunofluorescence mapping of type H vascular niches with topographically associated

RUNX2 + osteoprogenitors. Scale bar: 100 μm. (E, F) Co-localization of osteoprogenitor markers ALP + (E) /OPN + (F) and EMCN + type H vasculature within the bone defect region. Scale bar: 100 μm. *p < 0.05, **p < 0.01 (verse Control); ##p<0.01 (verse SRT1720); ※p<0.05, ※※p<0.01 (verse EX527) by one-way ANOVA with Tukey's post-hoc test.

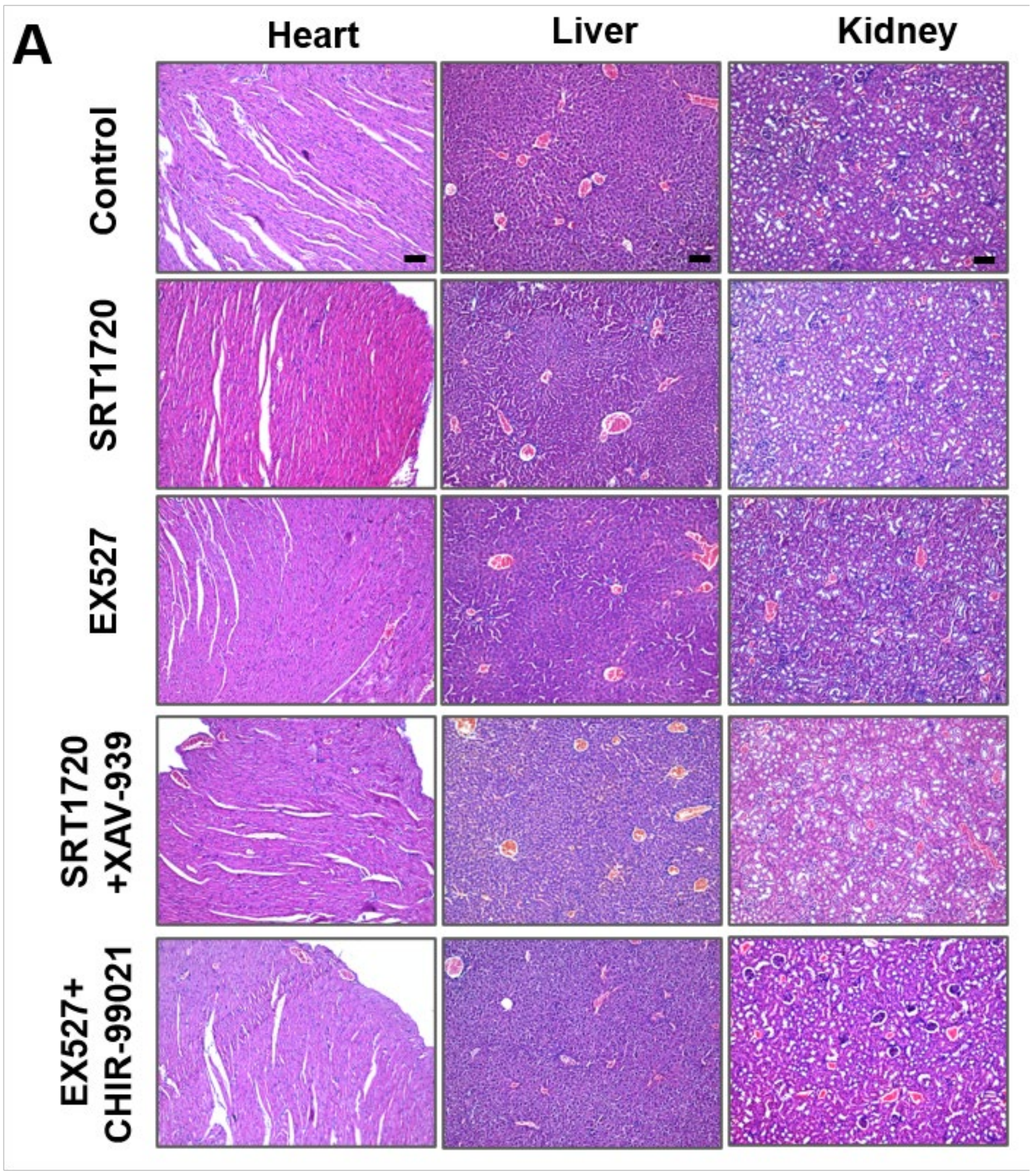

Figure S8 Systemic biosafety evaluation of pharmacological intervention through visceral histopathology, related to Figure 7. (A) Representative H&E-stained sections of cardiac, hepatic, and renal tissues from the experiments. Scale bar: 200 μm.

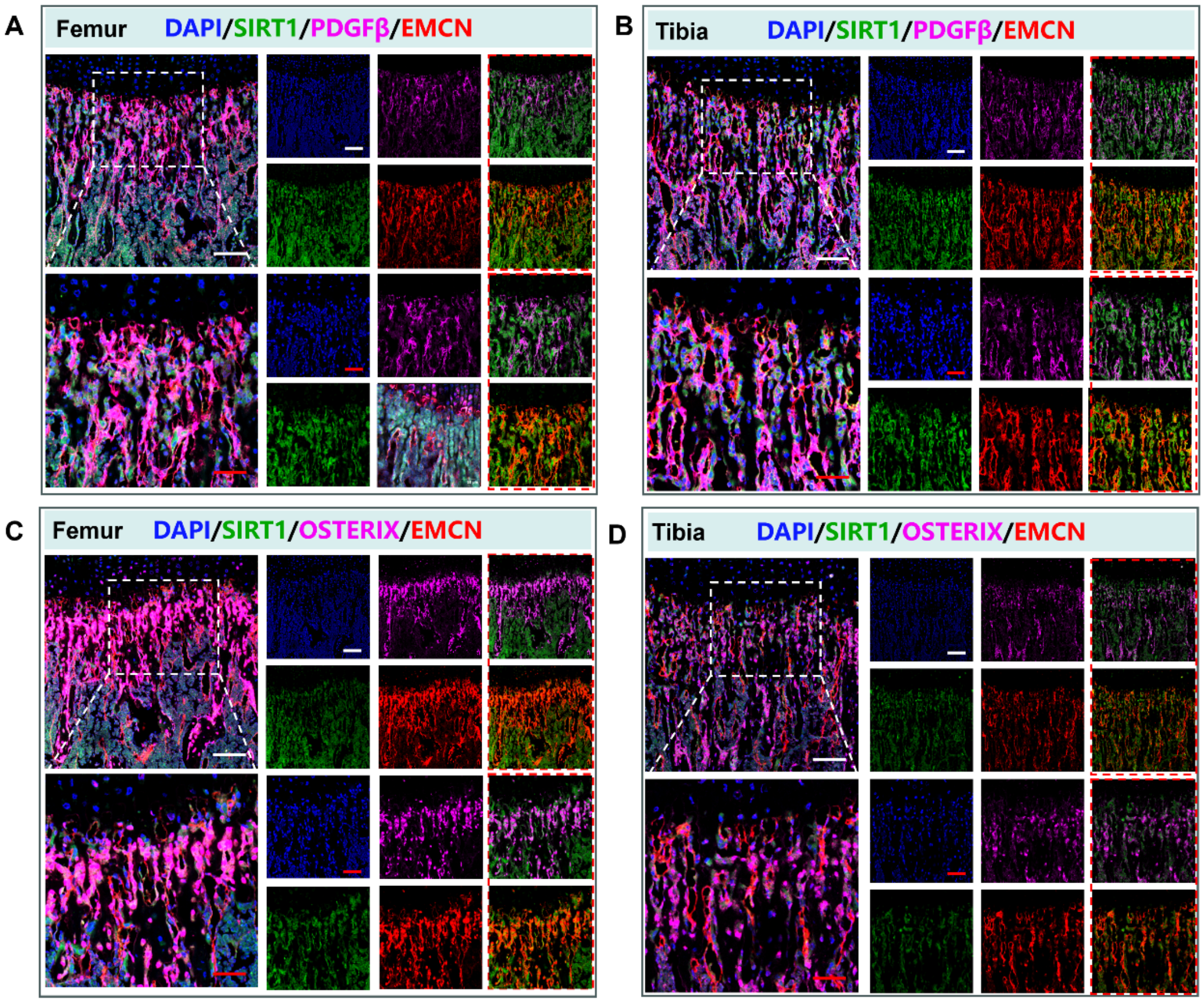

Figure S9. Distribution of SIRT1 in the bone microenvironment reveals widespread expression across endothelial, perivascular, and osteogenic populations, related to Discussion. (A-B) Images of the femur (A) and tibia (C) metaphysis from young mice stained for SIRT1, PDGFβ, and EMCN. Scale bars: 100 μm (white), 50 μm (red). (C-D) Images of the femur (C) and tibia (D) metaphysis from young mice stained for SIRT1, OSTERIX, and EMCN. Scale bars: 100 μm (white), 50 μm (red).

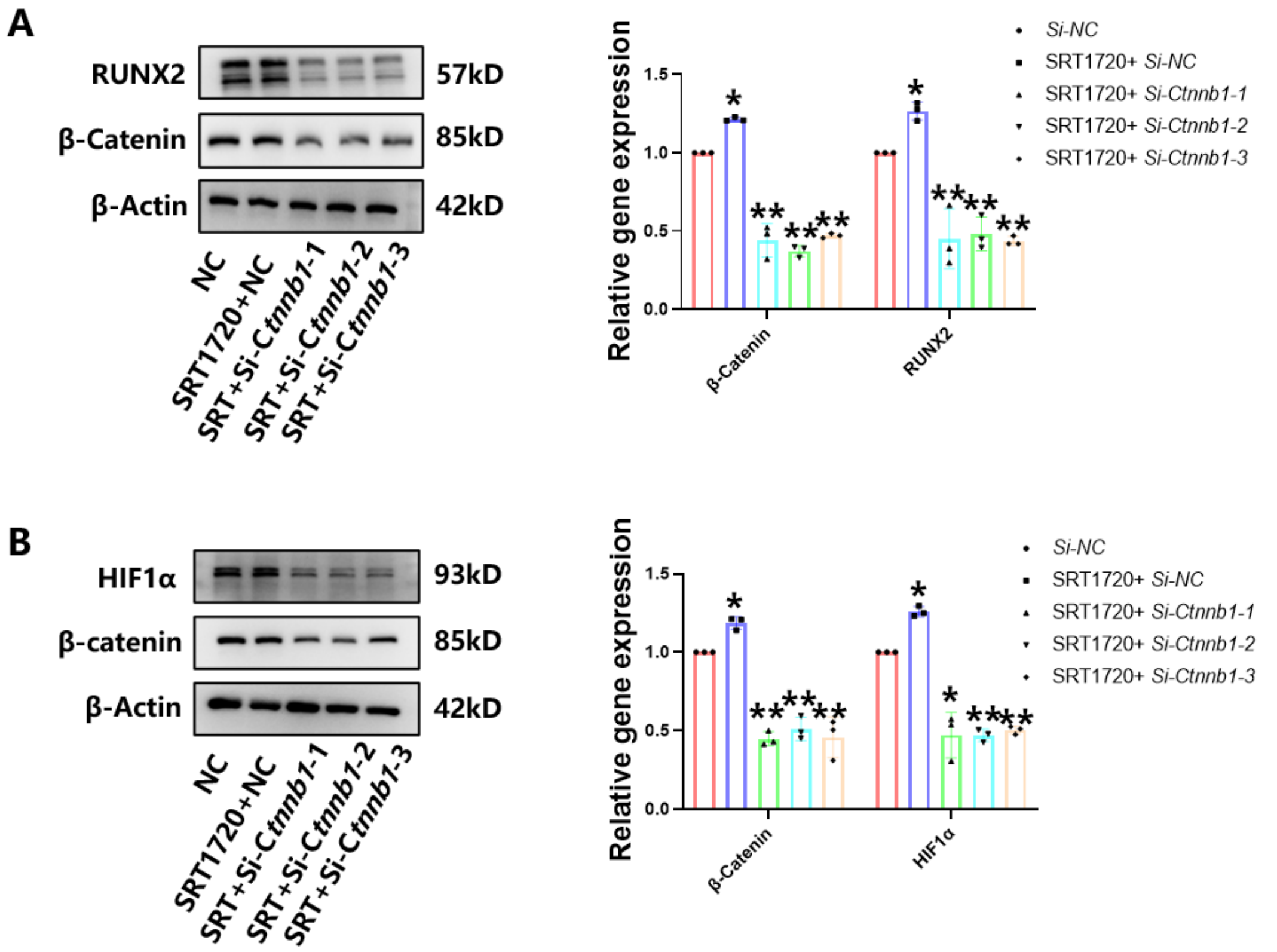

Figure S10 Three independent siRNAs targeting Ctnnb1 mRNA for β-catenin knockdown in senescent BMSCs and HUVECs, related to Discussion. (A) Protein expression of RUNX2, an osteogenic marker, in senescent BMSCs. (B) Protein expression of HIF1α, an angiogenic marker, in senescent HUVECs. Data presented as mean ± SD; $^{*}p < 0.05$, $^{**}p < 0.01$ by one-way ANOVA with Tukey's post-hoc test.

Supplemental tables

Table S1: Information of primer and siRNA sequences used in the experiment

Primer		
Gene	Forward Primer (5'-3')	Reverse Primer (5'-3')
Ctnnb1(Mouse)	GTCAGTGCAGGAGGCCGA	TGGCTGACAGCAGCTTTTCT
Ctnnb1(Homo)	CTGAGGAGCAGCTTCAGTCC	GGCCATGTCCAACTCCATCA
siRNA		
Gene	Sense (5'-3')	Antisense (5'-3')
scramble siRNA	*UUCUCCGAACGUGUCACGUTT*	*ACGUGACACGUUCGGAGAATT*
Ctnnb1 siRNA-1（Mouse）	*CCAGGUGGUAGUUAAUAAATT*	*UUUAUUAACUACCACCUGGTT*
Ctnnb1 siRNA-2（Mouse）	*CACCUCCCAAGUCCUUUAUTT*	*AUAAAGGACUUGGGAGGUGTT*
Ctnnb1 siRNA-2（Mouse）	*GGGUUCCGAUGAUAUAAAUTT*	*AUUUAUAUCAUCGGAACCCTT*
Ctnnb1 siRNA-1（Homo）	*GUCCUUCACUCAAGAACAATT*	*UUGUUCUUGAGUGAAGGACTT*
Ctnnb1 siRNA-2（Homo）	*GGACUCUGGAAUCCAUUCUTT*	*AGAAUGGAUUCCAGAGUCCTT*
Ctnnb1 siRNA-3（Homo）	*GGGAUGUUCACAACCGAAUTT*	*AUUCGGUUGUGAACAUCCCTT*
